# Mutual dependence of the MRTF–SRF and YAP–TEAD pathways in cancer-associated fibroblasts is indirect and mediated by cytoskeletal dynamics

**DOI:** 10.1101/gad.304501.117

**Published:** 2017-12-01

**Authors:** Charles T. Foster, Francesco Gualdrini, Richard Treisman

**Affiliations:** Signalling and Transcription Group, Francis Crick Institute, London NW1 1AT, United Kingdom

**Keywords:** transcription, MRTF, SRF, YAP, TEAD, mechanotransduction, cancer-associated fibroblast

## Abstract

Both the MRTF–SRF and the YAP–TEAD transcriptional regulatory networks respond to extracellular signals and mechanical stimuli. We show that the MRTF–SRF pathway is activated in cancer-associated fibroblasts (CAFs). The MRTFs are required in addition to the YAP pathway for CAF contractile and proinvasive properties. We compared MRTF–SRF and YAP–TEAD target gene sets and identified genes directly regulated by one pathway, the other, or both. Nevertheless, the two pathways exhibit mutual dependence. In CAFs, expression of direct MRTF–SRF genomic targets is also dependent on YAP–TEAD activity, and, conversely, YAP–TEAD target gene expression is also dependent on MRTF–SRF signaling. In normal fibroblasts, expression of activated MRTF derivatives activates YAP, while activated YAP derivatives activate MRTF. Cross-talk between the pathways requires recruitment of MRTF and YAP to DNA via their respective DNA-binding partners (SRF and TEAD) and is therefore indirect, arising as a consequence of activation of their target genes. In both CAFs and normal fibroblasts, we found that YAP–TEAD activity is sensitive to MRTF–SRF-induced contractility, while MRTF–SRF signaling responds to YAP–TEAD-dependent TGFβ signaling. Thus, the MRF–SRF and YAP–TEAD pathways interact indirectly through their ability to control cytoskeletal dynamics.

## Abstract (precis)

In this study, Foster et al. demonstrate that activation of the MRTF–SRF signaling pathway occurs in cancer-associated fibroblasts (CAFs) and is required for their proinvasive and contractile activity. The investigators also identify shared and specific direct genomic targets for MRTF–SRF and YAP–TEAD and show that MRTF and YAP are independently regulated by cytoskeletal dynamics and that this is the basis for their mutual dependence.

The interface between mechanical force and gene expression is central to our understanding of normal and transformed cell behavior (Schwartz 2010; DuFort et al. 2011). Variation in extracellular matrix (ECM) stiffness and cell adhesion can affect gene expression programs to promote alternative cell fate choices (Engler et al. 2006; Discher et al. 2009), while, in cancer, malignant progression of solid tumors is associated with increased stiffening of the tumor microenvironment (Paszek et al. 2005; Levental et al. 2009; Schedin and Keely 2011). Cancer-associated fibroblasts (CAFs) play an important role in cancer progression. These myofibroblast-like cells, which express high levels of αSMA, promote cancer cell growth, invasion, and metastasis by paracrine signaling and ECM remodeling (for review, see Kalluri 2016).

The MRTF and YAP transcriptional pathways contribute to the response to mechanical stress. The MRTFs—which are recruited to DNA by their partner, SRF—control expression of dozens of cytoskeletal genes, including αSMA (for review, see Olson and Nordheim 2010). They respond to Rho-GTPase signals, directly sensing changes in G-actin concentration via their regulatory RPEL domain, and accumulate in the nucleus when G-actin levels are low (Cen et al. 2003; Miralles et al. 2003; Vartiainen et al. 2007). The MRTFs can also be activated by substrate stiffness or direct integrin engagement (Zhao et al. 2007; Buxboim et al. 2014; Esnault et al. 2014)*.* YAP and TAZ, which bind DNA in association with members of the TEAD family of DNA-binding cofactors, were first characterized as effectors of the Hippo growth control pathway (for review, see Meng et al. 2016). In addition, they also respond to Rho-GTPase signaling, accumulating in the nucleus in response to high cytoskeletal tension induced by mechanical cues (Dupont et al. 2011; Wada et al. 2011; Das et al. 2016). Previous studies have shown that YAP is activated in CAFs and that YAP–TEAD signaling maintains their contractile and proinvasive properties (Calvo et al. 2013; Dupont 2016).

Several observations suggest that the MRTFs and YAP/TAZ may functionally interact even though they do not share a common DNA targeting factor. The multiplicity of cytoskeletal genes and components of the YAP–TAZ interactome among direct MRTF–SRF genomic targets suggests the possibility of indirect pathway cross-talk (Dupont et al. 2011; Esnault et al. 2014). Some genes, such as *Ctgf*, *Cyr61*, and *Ankrd1*, contain binding sites for both SRF and TEAD and thus represent shared targets (for example, see Latinkic et al. 1991; Zhang et al. 2011). Moreover, YAP/TAZ and the MRTFs can physically interact, although it remains unclear whether this allows their recruitment to DNA independently of their own DNA-binding partners (Speight et al. 2016; Yu et al. 2016; Kim et al. 2017).

Here we investigate the relationship between MRTF–SRF and YAP–TEAD signaling in CAFs and normal fibroblasts. We show that CAFs exhibit elevated MRTF activity, which is required for their contractile and proinvasive properties. Expression of YAP–TEAD genomic targets and expression of MRTF–SRF genomic targets are mutually dependent even when they are directly targeted by only one of the pathways. Finally, we show that activation of either pathway potentiates the activity of the other indirectly and that this depends on cytoskeletal dynamics.

## Results

### MRTF–SRF signaling is activated in CAFs

**Figure 1.**
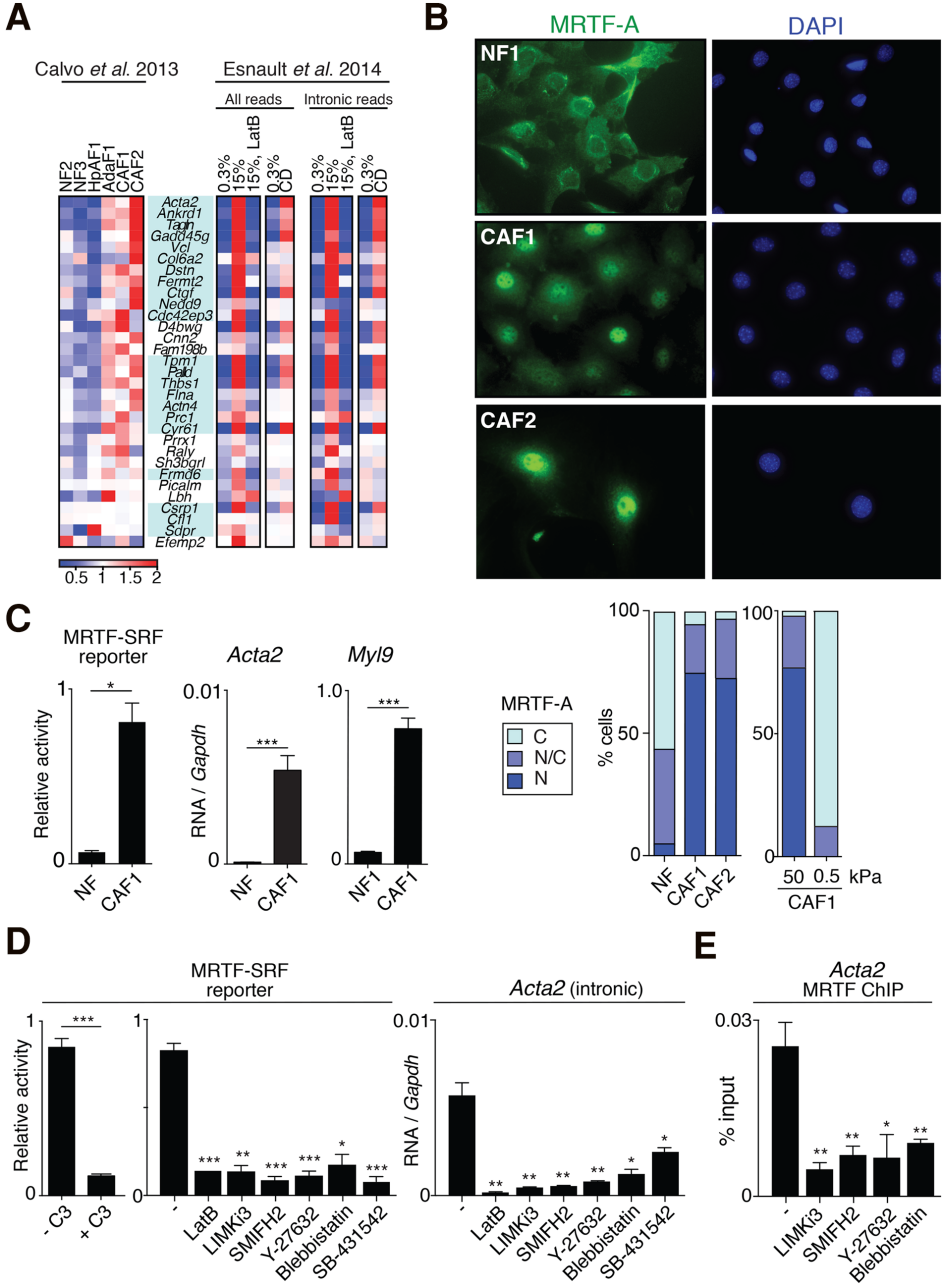
The MRTF–SRF pathway is activated in CAFs. (*A*) Heat map expression profiles of MRTF–SRF target genes (Esnault et al. 2014) that are overexpressed in CAFs (Calvo et al. 2013). (*Left*) Fibroblasts from various stages of the mouse PyMT mammary tumor model (Calvo et al. 2013). (*Right*) NIH3T3 fibroblasts stimulated either with serum; with or without latrunculin B (LatB), which inhibits MRTF activation; or with cytochalasin D (CD), which specifically activates the MRTFs by competing for G-actin binding (Vartiainen et al. 2007). Genes shaded in blue are cytoskeletal components or regulators. (*B*, *top*) Immunofluorescence microscopy of MRTF-A in normal mammary fibroblasts (NF1), CAF1, CAF2. (*Bottom*) MRTF-A localization in NFs and CAFs and in CAF1 cells plated on stiff and soft polyacrylamide hydrogels. (C) Higher cytoplasmic concentration; (N/C) equal concentration over the whole cell; (N) higher nuclear concentration. (*C*) Comparison of MRTF–SRF reporter gene activity and *Acta2* and *Myl9* transcripts in NF1 and CAF1 cells. Data are mean ± SEM. *n* = 3. (*) *P* < 0.05. (*D*) CAF1 cells were transfected with MRTF–SRF reporter and C3 transferase expression plasmids and treated with inhibitors as indicated before analysis of reporter activity or *Acta2* transcripts. Data are mean ± SEM. *n* = 3. (***) *P* < 0.001; (**) *P* < 0.01; (*) *P* < 0.05 by *t*-test. (*E*) ChIP analysis of MRTF-A in inhibitor-treated CAF1 cells. Data are mean ± SEM. (**) *P* < 0.01; (*) *P* < 0.05.

A previous analysis of stromal fibroblasts associated with tumors in the MMTV-PyMT mouse mammary carcinoma model suggested that expression of MRTF–SRF target genes may be increased in CAFs (Calvo et al. 2013). We compared that data set with our previous ChIP-seq (chromatin immunoprecipitation [ChIP] combined with high-throughput sequencing) analysis of MRTF–SRF target genes in serum-stimulated NIH3T3 fibroblasts (Esnault et al. 2014; Gualdrini et al. 2016). A substantial number of MRTF–SRF target genes, predominantly associated with cytoskeletal regulation, including the myofibroblast activation marker αSMA (*Acta2*), increased with severity of disease stage (Fig. 1A; Table 1A; Supplemental Table S1). The MRTF–SRF target gene data set also exhibited significant overlap with genes overexpressed in myofibroblastic CAFs associated with human pancreatic ductal adenocarcinoma (PDAC) (Table 1A; see the Discussion; Ohlund et al. 2017).

**Table 1.**
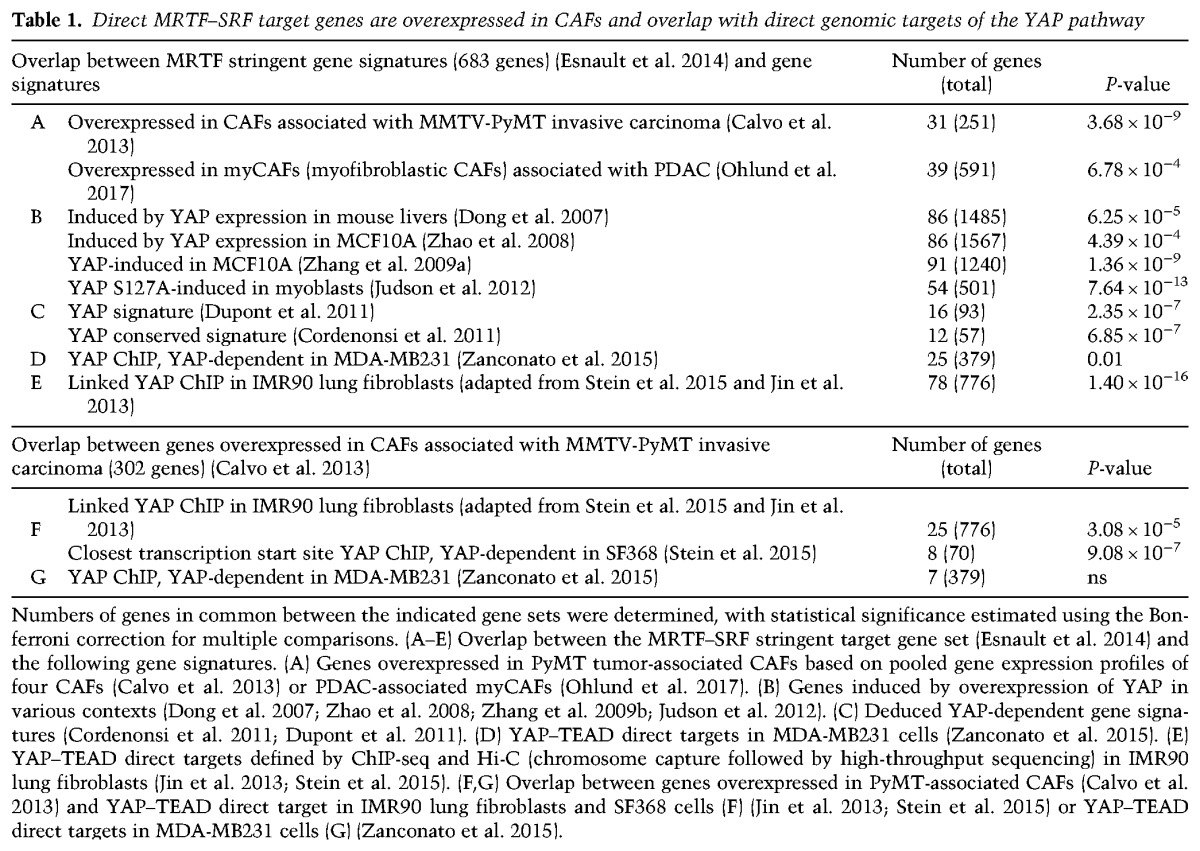
Direct MRTF–SRF target genes are overexpressed in CAFs and overlap with direct genomic targets of the YAP pathway

In fibroblasts, extracellular signaling through the Rho-actin pathway promotes MRTF nuclear accumulation (Miralles et al. 2003; Vartiainen et al. 2007). Although normal mammary fibroblasts (NFs) exhibited predominantly cytoplasmic and pancellular localization of MRTF-A in serum-starved conditions, the majority of CAF1 and CAF2 cells exhibited nuclear MRTF-A (Fig. 1B). Consistent with this, an MRTF–SRF reporter gene exhibited increased activity in CAFs compared with NFs (Fig. 1C) even though MRTF-A and MRTF-B expression was comparable in the two cell types (Supplemental Fig. S1A). CAFs exhibited elevated expression of the major myofibroblast and contractility markers αSMA/*Acta2* and MLC2/*Myl9* (Fig. 1C; Supplemental Fig. S1B). Both of these genes are MRTF targets, and their expression in CAFs was MRTF-dependent (Supplemental Fig. S1B). In contrast, the TCF (ternary complex factor)–SRF target gene *Egr1* was expressed at comparable levels in NFs and CAFs (Supplemental Fig. S1C). Consistent with these observations, ChIP analysis demonstrated increased MRTF-A, SRF, and RNA polymerase II (Pol II) recruitment at *Acta2* but not *Egr1* (Supplemental Fig. S1E).

Previous studies have shown that MRTF activation in serum-stimulated fibroblasts is RhoA-dependent and reflects depletion of the G-actin pool as a result of formin or LIM kinase activation (Sotiropoulos et al. 1999; Tominaga et al. 2000). Consistent with this, in CAFs, the elevated activity of *Acta2* and the transfected MRTF–SRF reporter was blocked when Rho was inactivated by C3 transferase expression; upon depolymerization of F-actin by latrunculin B (LatB); by the LIMK inhibitor LIMKi3; and by the formin inhibitor SMIFH2 (Fig. 1D). *Acta2* and reporter expression was also inhibited by treatment with the Rho-actin inhibitor CCG-203971 (Johnson et al. 2014; Supplemental Fig. S1D). These inhibitor treatments also reduced MRTF and SRF recruitment to *Acta2* as assessed by ChIP (Fig. 1E; Supplemental Fig. S1E).

The establishment and maintenance of the activated state of CAFs and myofibroblasts are promoted by substrate stiffness (for reviews, see Dupont 2016; Kalluri 2016) and TGFβ signaling (Huang et al. 2012; O'Connor et al. 2015), so we examined the role played by these stimuli in maintaining MRTF activation in CAFs. While MRTF-A remained nuclear when CAFs were plated on stiff (50-kPa) hydrogel substrates, it relocalized to the cytoplasm upon plating on soft (0.5-kPa) hydrogel (Fig. 1B; Supplemental Fig. S1F). Consistent with this, treatment of CAFs with Y27632 or blebbistatin, which impair actomyosin contractility, inhibited both *Acta2* expression and MRTF ChIP at *Acta2* (Fig. 1D,E). TGFβ signaling was elevated in CAFs, as assessed by levels of phosphorylated Smad2 (Supplemental Fig. S2A). Treatment of CAFs with the TGFβ family receptor kinase inhibitor SB-431542 abolished Smad2 phosphorylation and induced relocalization of MRTF-A to the cytoplasm (Supplemental Fig. S2B,C). SB-431542 treatment also reduced expression of the MRTF–SRF reporter gene and the MRTF–SRF target genes *Acta2* and *Myl9* (Fig. 1D; Supplemental Fig. S2C,D). Conversely, in NFs, TGFβ treatment induced MRTF-A nuclear accumulation (Supplemental Fig. S2E) and activated *Acta2* and *Myl9* in an MRTF-dependent fashion (Supplemental Fig. S2F). Taken together, these results establish that MRTF activation is a marker of the CAF activated state and show that TGFβ signaling and contractility are required to maintain it.

### The MRTFs are required for CAF contractility and matrix remodeling activity

**Figure 2.**
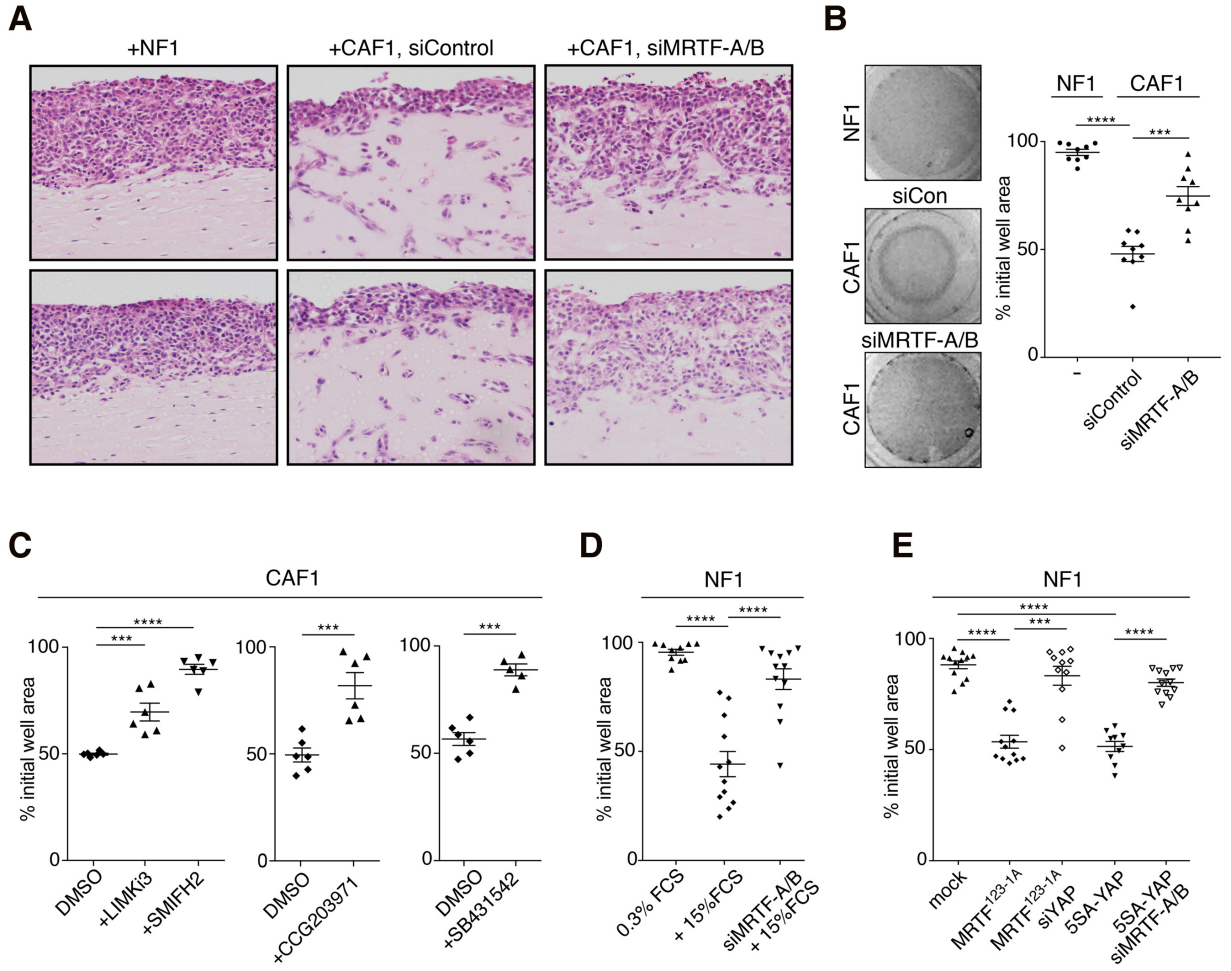
MRTFs are required for CAF matrix remodeling activity and contractility. (*A*) Representative images of invasion of 4T1 breast carcinoma cells into collagen–Matrigel containing NF1 or CAF1 cells treated with control or MRTF-A/B siRNA as indicated. (*B*,*C*) MRTF activity is required for contractility of CAF1 cells plated in collagen–Matrigel. (*B*) Cells were treated for 72 h with MRTF-A/B siRNA. *n* = 3, each plated into three gels. (*C*) Cells were pretreated for 20 h with inhibitors as indicated before plating. *n* = 3, each plated into two gels. (*D*,*E*) Normal fibroblasts exhibit MRTF- and YAP-stimulated contractility. (*D*) NF1 cells with siMRTF-A/B pretreatment as indicated were stimulated with 15% FCS. *n* = 4, each plated into four gels. (*E*) Cells transfected with constitutively active MRTF^123-1A^ or 5SA-YAP expression plasmids with siMRTF-A/B or siYAP as indicated. *n* = 3, each plated into four gels. Data are mean contraction at 24 h ± SEM. (****) *P* < 0.0001; (***) *P* < 0.001, *t*-test.

We next tested the role of MRTF–SRF activity in matrix remodeling behavior and contractility, hallmarks of CAF function (Kalluri 2016). As a result of their increased ECM remodeling activity, when embedded in collagen gels, CAFs greatly facilitate matrix invasion by 4T1 carcinoma cells (Gaggioli et al. 2007). Strikingly, the proinvasive activity of CAFs was substantially impaired upon siRNA-mediated depletion of the MRTFs (Fig. 2A), as was their ability to contract collagen matrices (Fig. 2B). CAF contractility was also inhibited by SMIFH2 and LIMKi3, which inhibit F-actin assembly and MRTF activation; the MRTF inhibitor CCG-203971; and SB-431542 (Fig. 2C). Thus, MRTF activation is required for the proinvasive and contractile properties of CAFs.

NFs were not contractile, but their contractility was activated by serum stimulation, and this was MRTF-dependent (Fig. 2D). Expression of a constitutively active derivative of MRTF-A, MRTF^123-1A^, was also sufficient to induce contractility (Fig. 2E). Previous studies have shown that the enhanced contractility and matrix remodeling activity of CAFs are dependent on the transcriptional coactivator YAP, depletion of its relative TAZ having no effect in this context (Calvo et al. 2013). Consistent with this, we found that expression of the constitutively active YAP derivative 5SA-YAP was also sufficient to induce contractility in NFs (Fig. 2E). Strikingly, however, siRNA depletion experiments showed that MRTF^123-1A^-induced contractility was dependent on YAP and vice versa (Fig. 2E). Taken together, these data suggest that the enhanced contractility and proinvasive character of CAFs requires both MRTFs and YAP and that their activities are mutually dependent.

### Definition of candidate MRTF- and YAP-specific direct target gene sets

Analysis of the relationship between MRTF- and YAP-dependent gene expression requires the definition of direct genomic targets for each pathway. Ideally, such definition requires identification of genomic binding sites for the factor concerned and a demonstration that transcription of genes that are in close proximity to these sites is dependent on the factor itself or the signal pathway that regulates it. Previously, we used this approach to define MRTF–SRF target genes in mouse NIH3T3 fibroblasts based on transcriptional response to MRTF-linked signals and proximity to MRTF–SRF genomic binding sites (Esnault et al. 2014; Gualdrini et al. 2016). The MRTF–SRF gene signature overlaps significantly with genes overexpressed in CAFs (Table 1A) as well as genes induced by YAP overexpression in different cell types (Table 1B,C; Dong et al. 2007; Zhao et al. 2008; Zhang et al. 2009a; Cordenonsi et al. 2011; Dupont et al. 2011; Judson et al. 2012).

Recent ChIP-seq studies have identified genomic binding sites for YAP–TEAD in normal and transformed human cells (Stein et al. 2015; Zanconato et al. 2015), many of which are associated with genes that are MRTF–SRF targets in fibroblasts (Table 1D; Esnault et al. 2014; Gualdrini et al. 2016). We further refined this analysis by integrating the IMR90 ChIP-seq data with an IMR90 Hi-C (chromosome capture followed by high-throughput sequencing) data set (Jin et al. 2013) to identify genes whose transcription start sites (TSSs) are in physical contact with YAP–TEAD-binding sites. This analysis defined 776 potential YAP–TEAD direct targets in IMR90 fibroblasts (Supplemental Table S2B). Both the MRTF–SRF and the YAP–TEAD target gene sets are enriched for cytoskeletal and ECM remodeling genes and many genes involved in proliferation, signaling, and transcription (Supplemental Table S3). Many were also overexpressed in CAFs from the mouse PyMT mammary and KRas/p53 pancreatic cancer models (Calvo et al. 2013; Ohlund et al. 2017) and from human pancreatic, oral, and breast carcinoma (Table 1F,G; Supplemental Tables S4–S7; Farmer et al. 2009; Lim et al. 2011; Moffitt et al. 2015; Ohlund et al. 2017).

### MRTF- and YAP-specific direct target genes are activated in CAFs

**Figure 3.**
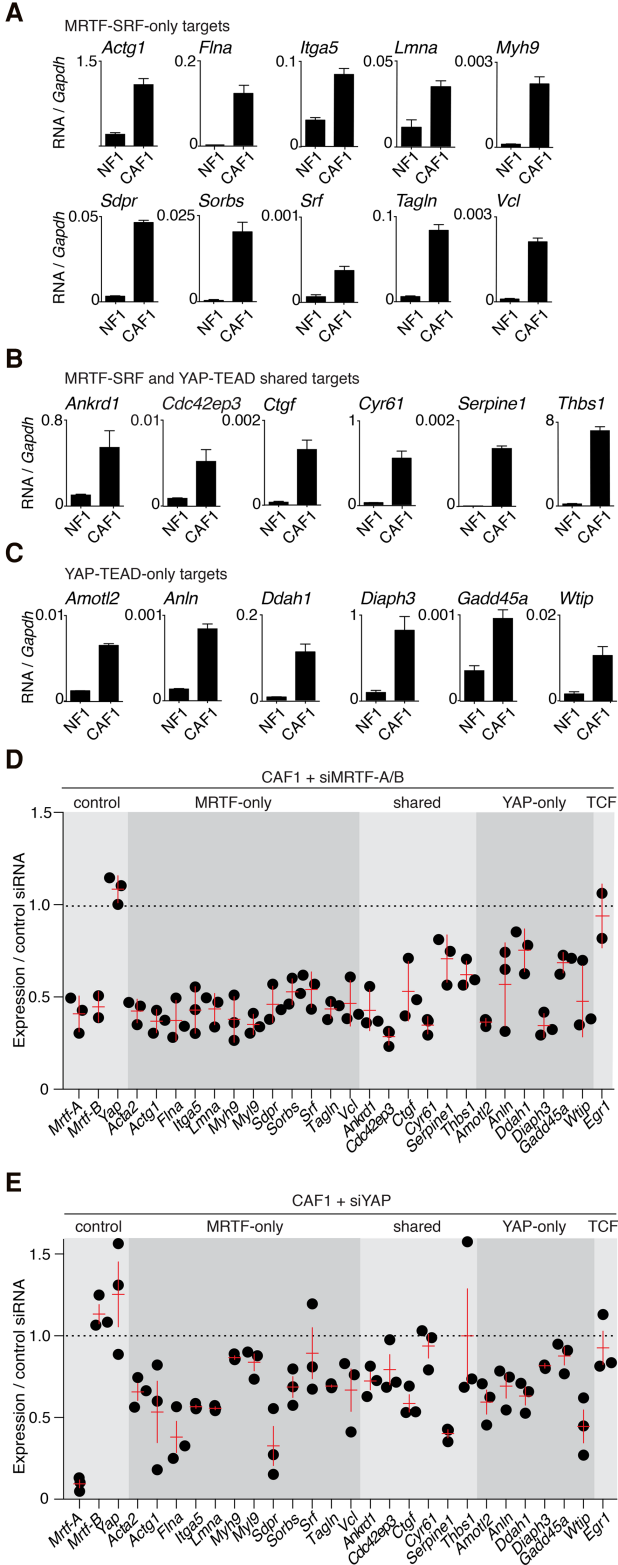
MRTF and YAP direct target genes are activated in CAFs. (*A*–*C*) Quantitative PCR (qPCR) analysis of MRTF and YAP target gene expression in NF1 and CAF1. Data are mRNA normalized to *Gapdh* transcripts. Mean ± SEM. *n* = 3. (*A*) MRTF-only targets. (*B*) Shared MRTF/SRF and YAP/TEAD targets. (*C*) YAP-only targets. (*D*,*E*) Mutual dependence of MRTF and YAP target gene expression. RNA from CAF1 cells was treated with siRNA against MRTF-A/B (*D*) or YAP (*E*). Data points represent independent siRNA treatments normalized to the geometric mean from triplicate control siRNA treatments. Red lines indicate mean and SEM.

Many of the YAP–TEAD target genes in human IMR90 cells were identified previously as direct MRTF–SRF target genes in mouse NIH3T3 cells (Table 1E; Supplemental Table S2E). Comparison of the two data sets thus allowed us to identify potential candidate direct target genes specific for MRTF–SRF alone, specific for YAP–TEAD alone, or shared by both regulators (referred to here as “MRTF-only,” “YAP-only,” and “shared” targets) (Supplemental Table S2C–E). All MRTF-only direct genomic targets analyzed were expressed at high levels in CAFs compared with NFs (Fig. 3A); we note that many of these were not detected as elevated in the previous analysis (Calvo et al. 2013), presumably because of the relatively insensitive Illumina array technique used. Similarly, expression of shared MRTF–SRF/YAP–TEAD genomic targets was also elevated in CAFs compared with NFs (Fig. 3B), as was expression of candidate YAP-only genomic targets (Fig. 3C). Strikingly, however, MRTF depletion impaired not only the expression of the MRTF-only genomic targets but also the YAP-only targets (Fig. 3D), and, conversely, YAP depletion impaired expression of MRTF–SRF genomic targets regardless of whether they contain YAP–TEAD-binding sites (Fig. 3E). Thus, MRTF–SRF signaling and YAP–TEAD signaling are mutually dependent.

### MRTF–SRF activity is dependent on YAP–TEAD and vice versa

**Figure 4.**
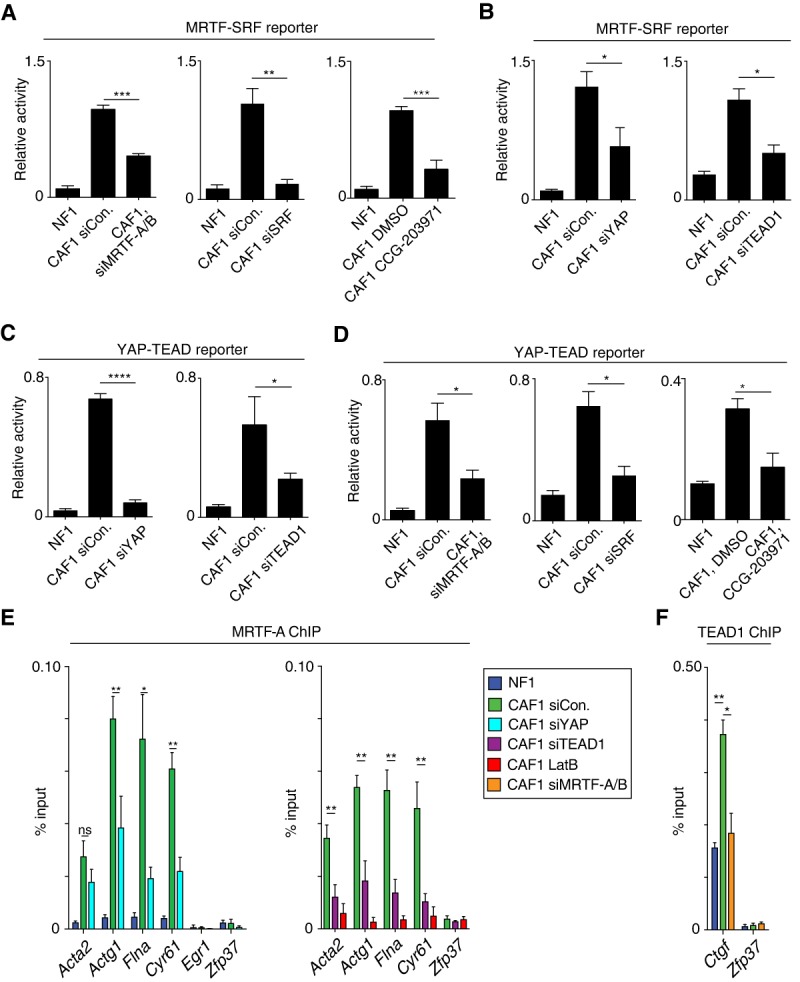
MRTF–SRF reporter gene activity is indirectly dependent on YAP–TEAD function and vice versa. NF1 or CAF1 cells were depleted of MRTFs, SRFs, YAP, or TEAD and transfected with the MRTF–SRF or YAP–TEAD reporters with CCG-203971 treatment and serum stimulation as indicated. Data are means ± SEM. *n* = 4. (****) *P* < 0.0001; (***) *P* < 0.001; (**) *P* < 0.01; (*) *P* < 0.05, by *t*-test. (*A*,*B*) Elevated MRTF–SRF reporter activity in CAFs. (*C*,*D*) Elevated YAP–TEAD reporter activity in CAFs. Note that YAP knockdown alone is sufficient to reduce reporter activity to baseline, suggesting that TAZ does not play a significant role in this system, consistent with previous functional analysis (Calvo et al. 2013). (*E*,*F*) ChIP analysis of MRTF-A binding (*E*) and TEAD1 binding (*F*). Controls were *Egr1* (TCF-SRF target, no MRTF-binding) and *Zfp37* (no SRF or TEAD binding). Cells were treated with siRNAs or LatB as indicated. (**) *P* < 0.01; (*) *P* < 0.05, by *t*-test.

The mutual dependence of MRTF–SRF and YAP–TEAD target gene expression even at those targets that are directly bound by only one of the transcription factor pairs strongly suggests that their mutual dependence reflects the influence of each on the other's cognate signaling pathway. To examine their mutual dependence more directly, we studied the regulation of MRTF–SRF and YAP–TEAD reporter genes. The elevated activity of an MRTF–SRF reporter in CAFs was sensitive to depletion of not only the MRTFs or SRFs and the Rho-actin inhibitor CCG-203971 (Fig. 4A) but also both YAP and its DNA-binding cofactor, TEAD1 (Fig. 4B). Similarly, a YAP–TEAD reporter also exhibited elevated activity in CAFs, which was dependent both on YAP and TEAD and on MRTF and SRF and was inhibited by CCG-203971 (Fig. 4C,D). Furthermore, MRTF and YAP were also mutually dependent in NFs. Serum-stimulated activity of the MRTF–SRF reporter was partially inhibited by depletion of YAP (Supplemental Fig. S3A), as was induction of the MRTF–SRF direct target genes *Acta2* and *Tagln* (Supplemental Fig. S3B,C). Conversely, the modest response to serum stimulation of the YAP–TEAD reporter and the YAP–TEAD-specific direct target genes *Amotl2* and *Ddah1* was inhibited by depletion of both MRTFs and YAP (Supplemental Fig. S3D–F).

We used ChIP to investigate whether the reporter results reflected changes in recruitment of MRTF and YAP to target genes. The enhanced recruitment of MRTF to MRTF–SRF target genes was enhanced in CAFs compared with NFs (Fig. 4E). It was significantly reduced upon depletion of either YAP or its DNA-binding partner protein, TEAD1, suggesting that MRTF activity is dependent on YAP–TEAD target gene expression (Fig. 4E). Inadequate antibodies precluded analysis of recruitment of YAP to YAP–TEAD targets in CAFs, but binding of its DNA-binding partner, TEAD1, was increased in CAFs compared with NFs and sensitive to depletion of MRTF (Fig. 4F). These data suggest that YAP–TEAD binding is MRTF-dependent. We confirmed this using human MDA-MB231 breast carcinoma cells, which are also migratory and invasive and display nuclear MRTF (Medjkane et al. 2009). Here, ChIP analysis revealed specific recruitment of MRTF-A to SRF sites and of YAP to TEAD sites; again, binding of MRTF was YAP-dependent and vice versa (Supplemental Fig. S4). Taken together, these results suggest that the mutual dependence of MRTF and YAP activity arises indirectly and is likely to reflect their influence on each other's signal pathway.

### MRTF and YAP contribute independently to target gene regulation in NFs and CAFs

Recent studies have demonstrated physical interaction between MRTF and YAP independent of their DNA-binding cofactor proteins, SRF and TEAD (Speight et al. 2016; Yu et al. 2016; Kim et al. 2017). The ChIP data presented above and the fact that YAP–TEAD and MRTF–SRF ChIP-seq data exhibit only partial overlap suggest that YAP–MRTF interaction is not sufficient to explain the mutual dependence of the two systems. Nevertheless, we sought to demonstrate directly that the two pathways are independently regulated, focusing on their response to the actin-binding drugs cytochalasin D (CD) and LatB. CD directly activates the MRTFs by blocking their inhibitory interaction with G-actin, while LatB inhibits MRTF activity by increasing cellular G-actin level (Miralles et al. 2003; Vartiainen et al. 2007), but both agents inhibit YAP by inhibiting F-actin assembly (Wada et al. 2011; Yu et al. 2012).

**Figure 5.**
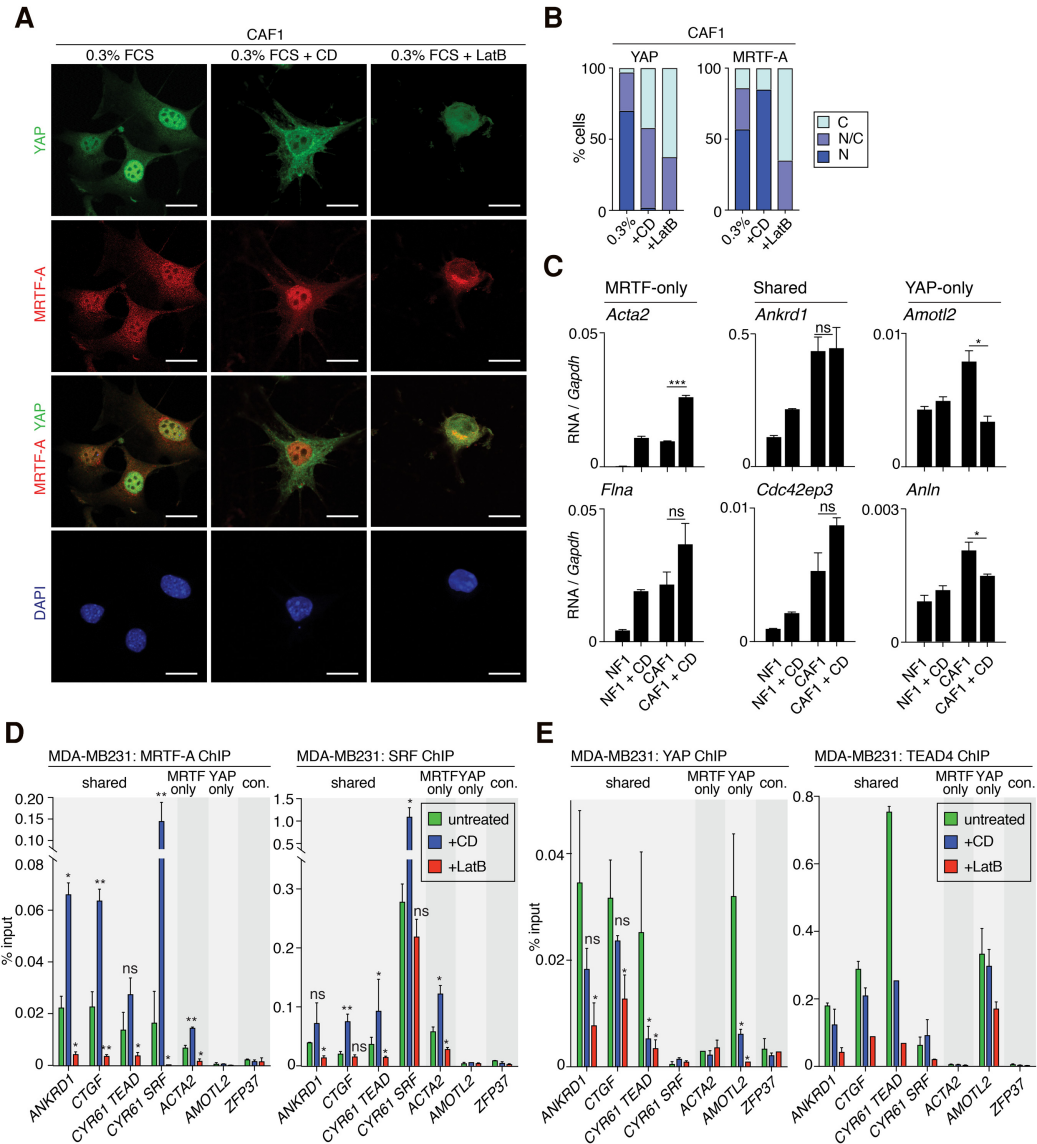
MRTF and YAP are independently regulated. (*A*,*B*) Immunofluorescence analysis of MRTF-A and YAP in cells treated with 2 µM CD, 1 µM LatB, or DMSO vehicle for 30 min. (*A*) Representative images. Bar, 20 µm. (*B*) Quantitation of YAP and MRTF-A subcellular localization from 20 fields of view at 20× magnification. (C) High cytoplasmic concentration; (N/C) equal concentration over the whole cell; (N) high nuclear concentration. (*C*) qPCR analysis of MRTF and YAP target gene intronic RNA in NF1 and CAF1 cells stimulated with CD for 30 min. Data are means ± SEM. *n* = 3. (*Left*) MRTF-only target genes. (*Middle*) Shared targets. (*Right*) YAP-only targets. (*D*) ChIP analysis of MRTF–SRF and YAP–TEAD recruitment to target genes in MDA-MB231 cells treated with CD or LatB for 30 min, as indicated. For target gene details, see Supplemental Figure S4. The *Zfp37* control does not bind either MRTF–SRF or YAP–TEAD. (*D*) MRTF-A and SRF binding. (*E*) YAP and TEAD4 binding. Data are means ± SEM. *n* = 3 independent chromatin preparations. (**) *P* < 0.01; (*) *P* < 0.05 Student's *t*-test, relative to untreated. The sensitivity of SRF binding to drug treatments likely reflects cooperative MRTF–SRF recruitment to promoters (Esnault et al. 2014; Gualdrini et al. 2016); the sensitivity TEAD4 binding to drug treatment is consistent with the YAP dependence of TEAD binding observed previously by others (Stein et al. 2015) and may reflect either cooperative recruitment or nuclear export of TEAD4.

In CAFs, LatB treatment resulted in the redistribution of both factors to the cytoplasm; in contrast, while CD treatment induced cytoplasmic localization of YAP, it potentiated nuclear accumulation of MRTFs (Fig. 5A,B). Consistent with this, the different classes of MRTF and YAP target genes responded differentially to CD treatment. In normal fibroblasts, MRTF-only and shared targets were CD-inducible, while YAP-only targets were not (Fig. 5C; Supplemental Fig. S5). In CAF1 cells, activity of MRTF-only targets was similar to that seen in CD-treated NFs and was further potentiated by CD (Fig. 5C; Supplemental Fig. S5A). The basal activity of shared MRTF–SRF/YAP–TEAD target genes in CAFs was higher than in CD-stimulated NFs, and CD stimulation had a modest or no further effect (Fig. 5C, middle; Supplemental Fig. S5B). In contrast, although YAP-only target genes were expressed at elevated levels in CAFs, their transcription was suppressed by CD treatment (Fig. 5C; Supplemental Fig. S5B).

Taken together, these results suggest that MRTF recruitment and YAP recruitment to DNA are independently regulated. Although our inability to detect mouse YAP by ChIP precluded a direct test of this model using CAFs, ChIP analysis in the human MDA-MB231 cell system corroborated the transcription data. At all target types, MRTF and YAP ChIP signals were abolished by LatB (Fig. 5D,E). However, CD treatment increased recruitment of MRTF and SRF to target genes (Fig. 5D) but decreased binding of YAP and TEAD (Fig. 5E). Thus, although the two factors can physically interact, their recruitment to DNA is independently regulated, and, at shared targets, expression levels represent the integration of separate signals at discreet MRTF–SRF- and YAP–TEAD-binding sites.

### Constitutively active MRTF and YAP derivatives are not mutually dependent

The data presented so far suggest that the mutual dependence of MRTF and YAP may reflect their ability to influence each other's upstream signal pathways, both of which involve alterations in cytoskeletal dynamics. To test this idea, we asked whether constitutively active derivatives of MRTF and YAP, whose activity is uncoupled from upstream signal pathways, are mutually dependent. MRTF^123-1A^ is nuclear and active because it cannot bind G-actin (Vartiainen et al. 2007), while 5SA-YAP lacks the phosphorylation sites required for its cytoplasmic retention by 14-3-3 protein (Wada et al. 2011; Zhao et al. 2012).

**Figure 6.**
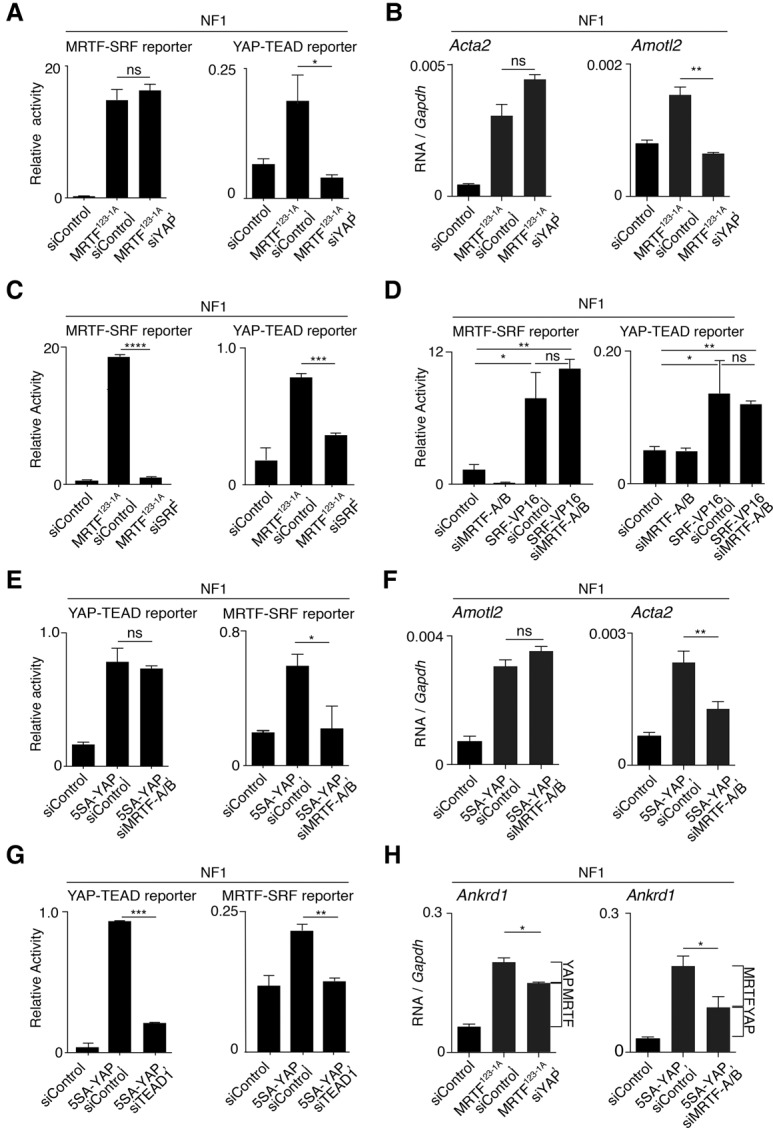
Constitutively active MRTF and YAP derivatives are not mutually dependent. NF1 cells were depleted of MRTFs, SRFs, YAP, or TEAD, as indicated, and transfected with the MRTF–SRF reporter or YAP–TEAD reporters together with expression plasmids encoding MRTF^123-1A^, SRF-VP16, or 5SA-YAP, as indicated. Data are means ± SEM. *n* = 3. (****) *P* < 0.0001; (***) *P* < 0.001; (**) *P* < 0.01; (*) *P* < 0.05, by Student's *t*-test. (*A*,*B*) MRTF^123-1A^ activates the MRTF–SRF reporter (*A*) and MRTF–SRF target genes (*B*) independently of YAP. (*C*) MRTF^123-1A^ requires SRF to activate both the MRTF–SRF and YAP–TEAD reporters. (*D*) SRF-VP16 does not require MRTFs to activate the MRTF–SRF and YAP–TEAD reporters. (*E*,*F*) 5SA-YAP activates the YAP–TEAD reporter (*E*) and YAP–TEAD target genes (*F*) independently of MRTFs. (*G*) 5SA-YAP requires TEAD1 to activate both the MRTF–SRF and YAP–TEAD reporters. (*H*) Indirect YAP and MRTF signaling contributes to activation of the MRTF–SRF and YAP–TEAD shared target gene *Ankrd1* by constitutively active MRTF^123-1A^ or 5SA-YAP. Notional contributions of YAP and MRTF activation are indicated by brackets.

In normal fibroblasts, expression of MRTF^123-1A^ effectively activated an MRTF–SRF reporter gene and MRTF-only target genes independently of YAP, consistent with a model in which transcription activation by MRTF does not require direct recruitment of YAP (Fig. 6A; Supplemental Fig. S6A). Activation of both MRTF–SRF and YAP–TEAD reporters by MRTF^123-1A^ was dependent on SRF (Fig. 6C), and both reporters could also be activated by expression of the constitutively active SRF derivative SRF-VP16 independently of MRTF (Fig. 6D). In contrast, MRTF^123-1A^ also activated the YAP–TEAD reporter gene and endogenous YAP–TEAD targets, but this was YAP-dependent (Fig. 6A,B; Supplemental Fig. S6B). Together, these data strongly suggest that MRTF^123-1A^ activates the YAP–TEAD pathway by inducing MRTF–SRF target gene expression.

Similarly, although the constitutively active YAP derivative 5SA-YAP activated both the YAP–TEAD reporter and YAP–TEAD target genes independently of MRTF (Fig. 6E,F; Supplemental Fig. S7A), its ability to activate the MRTF–SRF reporter was dependent on TEAD (Fig. 6G; Supplemental Fig. S7B). In contrast, activation of shared target genes by MRTF^123-1A^ and 5SA-YAP was partially dependent on YAP and MRTF, respectively (Fig. 6H; Supplemental Figs. S6C, S7C). These data are consistent with our assignment of the various target genes to MRTF-only, YAP-only, and shared classes. Moreover, they suggest that constitutively active MRTF activates YAP upstream signal pathways as a result of its ability to activate MRTF–SRF target gene expression and vice versa.

### MRTF–SRF signaling activates YAP through cell contractility

**Figure 7.**
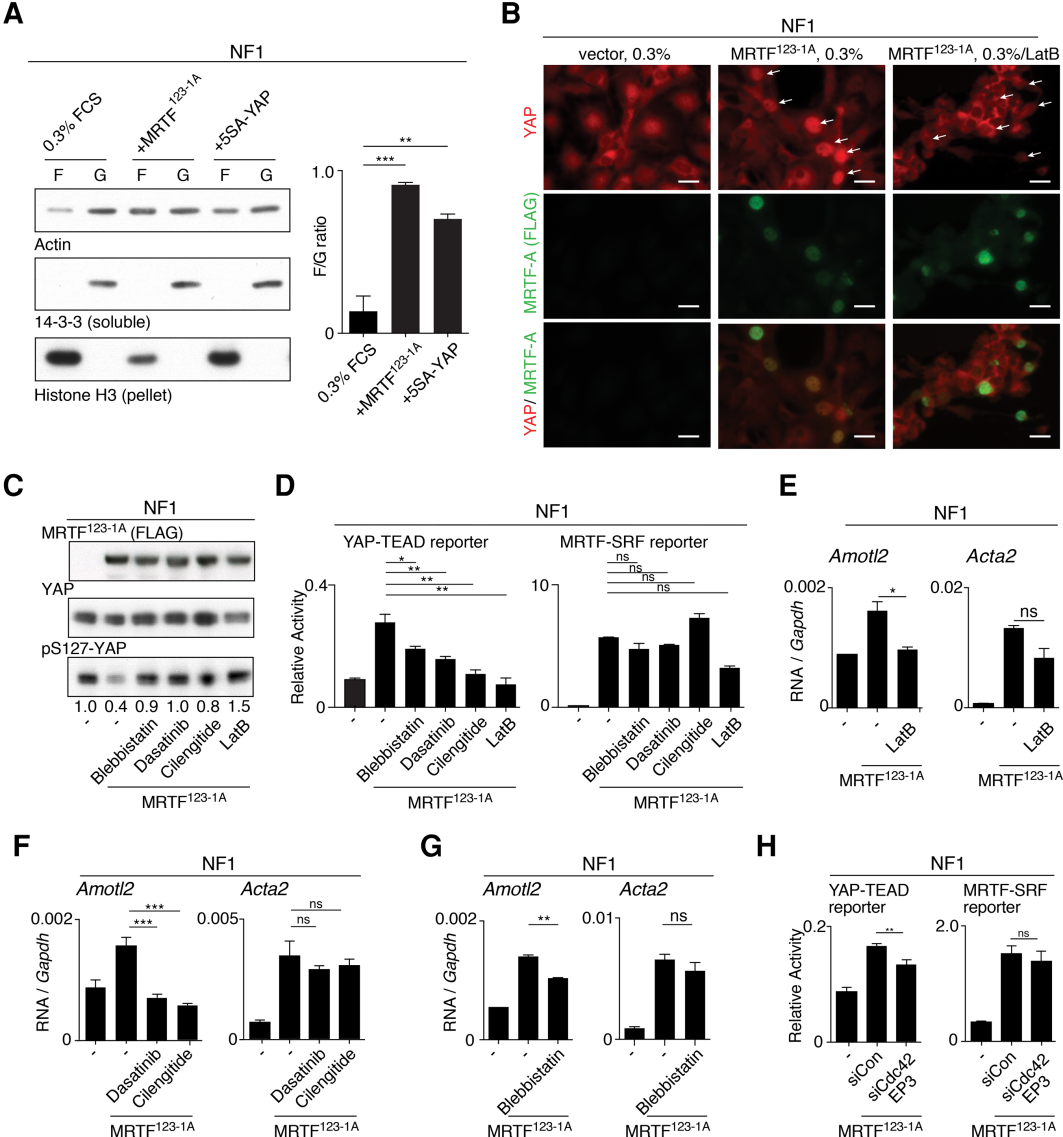
MRTF activates YAP through cell contractility. (*A*, *left*) Constitutively active MRTF^123-1A^ and 5SA-YAP increase the proportion of actin in the pellet (lanes *F*) compared with soluble fractions (lanes *G*) in sedimentation assays. 14-3-3 and H3 are controls for soluble and pellet fractions. (*Right*) Quantitation. Data are means ± SEM. *n* = 3. (**) *P* < 0.01, Student's *t*-test. (*B*) Immunofluorescence analysis of NF1 cells with or without MRTF^123-1A^ expression with 30 min LatB treatment, as indicated. Arrows indicate cells overexpressing MRTF^123-1A^. Bar, 25 µm. (*C*) Immunoblot analysis of YAP S127 phosphorylation in NF1 cells expressing MRTF^123-1A^ with 2-h inhibitor treatments, as indicated. (*D*) Analysis of MRTF–SRF or YAP–TEAD reporter activity in cells expressing MRTF^123-1A^ with inhibitor treatment, as indicated. Data are means ± SEM. *n* = 3. (**) *P* < 0.01; (*) *P* < 0.05. (*E*–*G*) Cells expressing MRTF^123-1A^ were treated with inhibitors as indicated, and expression of intronic RNA of the MRTF-only target *Acta2* and the YAP-only target *Amotl2*. Data are means ± SEM. *n* = 3. (***) *P* < 0.001; (**) *P* < 0.01; (*) *P* < 0.05. (*H*) Analysis of reporter activity in NF1 cells expressing MRTF^123-1A^ with depletion of Cdc42EP3, as indicated. Analysis was as in *D*.

We next investigated the mechanistic basis for the mutual dependence of MRTF–SRF and YAP–TEAD signaling. Both pathways are responsive to the state of actin dynamics, so we first investigated whether MRTF^123-1A^ or 5SA-YAP affects F-actin assembly. Both proteins promoted F-actin assembly in NFs, as judged by F-actin pelleting assays and phalloidin staining (Fig. 7A; Supplemental Fig. S8A). Expression of MRTF^123-1A^ in NFs induced YAP nuclear accumulation, which was inhibited upon depolymerization of F-actin by LatB (Fig. 7B). Consistent with this, in MDA-MB231 cells, MRTF^123-1A^ expression promoted increased YAP ChIP at YAP targets, which was abolished by LatB (Supplemental Fig. S8C).

Cell adhesion and cell contractility act via Src kinases to activate YAP by antagonizing LATS-mediated phosphorylation of YAP S127, a major target for Hippo signaling (Kim and Gumbiner 2015; Si et al. 2017; for review, see Meng et al. 2016). Cell adhesion, actomyosin contractility, and Src kinases are also required for maintenance of active YAP in CAFs (Calvo et al. 2013). Expression of MRTF^123-1A^ in NF1 cells decreased YAP S127 phosphorylation, which was accompanied by increased Src S416/418 and MLC2 phosphorylation (Supplemental Fig. S8D). In NF1 cells, inhibition of myosin (blebbistatin), Src family kinases (dasatinib), or integrinαv (cilengitide) blocked MRTF^123-1A^-induced YAP S127 dephosphorylation (Fig. 7C) and inhibited activation of the YAP reporter by MRTF^123-1A^ without affecting activation of the MRTF reporter (Fig. 7D). Activation of YAP-only and MRTF-only target genes by MRTF^123-1A^ was affected in a similar manner (Fig. 7E–G).

These results show that YAP activation by MRTF^123-1A^ involves increased cell contractility, a property that is strongly dependent on MRTF–SRF target gene expression (Esnault et al. 2014; Gualdrini et al. 2016). We showed above that in NFs, MRTF^123-1A^ activates expression of contractile genes—including αSMA/A*cta2*, MLC2/*Myl9*, and *Myh9* (Fig. 6B; Supplemental Fig. S6)—and contractility itself (Fig. 2E), and, in CAFs, contractile gene expression and contractility are MRTF-dependent (Fig. 2B; Supplemental Fig. S1B). Expression of integrins *Itgb3* and *Itgav*, specific targets for cilengitide action, was elevated in CAFs and could be potentiated in NFs by MRTF^123-1A^ expression (Supplemental Fig. S8D,E).

Recent studies have shown that the septin regulator Cdc42EP3 is required in CAFs for F-actin assembly, contractility, and YAP activation (Calvo et al. 2015). In CAFs, expression of Cdc42EP3, which is a direct MRTF–SRF target in fibroblasts (Esnault et al. 2014; Gualdrini et al. 2016), was elevated and MRTF-dependent (Fig. 3D), and, in NFs, it was strongly activated by MRTF^123-1A^ and CD (Fig. 5C; Supplemental Fig. S6C). Consistent with the reported dependence of F-actin assembly on Cdc42EP3 levels (Calvo et al. 2015), the ability of MRTF^123-1A^ to induce activity of the YAP–TEAD reporter and YAP–TEAD target genes in NFs was sensitive to Cdc42EP3 depletion (Fig. 7H). Taken together with the preceding results, these data show that MRTF–SRF signaling potentiates YAP activity through activation of multiple genes involved in cell contractility.

**Figure 8.**
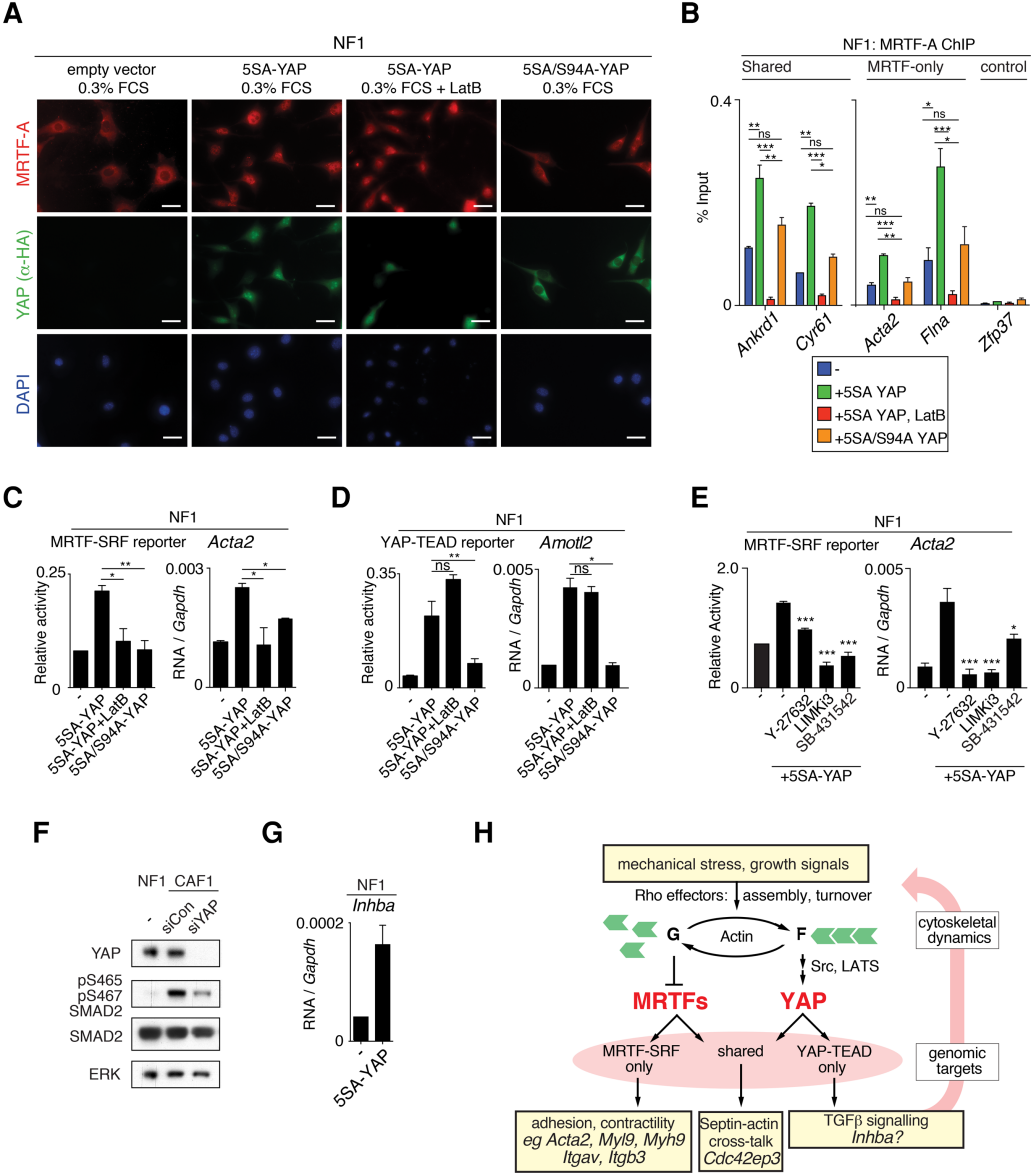
YAP–TEAD activation of MRTF requires TGF-β signaling. (*A*) Immunofluorescence analysis of NF1 cells expressing 5SA-YAP or 5SA/S94A-YAP with LatB treatment, as indicated. (*B*) ChIP analysis of NF1 cells expressing 5SA-YAP or 5SA/S94A-YAP before or after 30 min of LatB treatment. Data are mean ± SEM. (***) *P* < 0.001; (**) *P* < 0.01; (*) *P* < 0.05. (*C*–*E*) Cells were transfected with the MRTF–SRF reporter and 5SA-YAP or 5SA/S94A-YAP and treated with the indicated inhibitors. Data are mean ± SEM. *n* = 3. (**) *P* < 0.01; (*) *P* < 0.05. (*C*) Activation of the MRTF reporter and MRTF-only target *Acta2* is sensitive to LatB. (*D*) Activation of the YAP reporter and YAP-only target *Amotl2* is not sensitive to LatB. (*E*) MRTF–SRF reporter activity or *Acta2* expression requires F-actin assembly and TGFβ signaling. (*F*) Immunoblot analysis of YAP, S465/S467-diphosphorylated Smad2, total Smad2, and ERK control in NF1 and CAF1 cells with or without depletion of YAP. (*G*) *Inhba* expression levels in NF1 cells with or without 5SA-YAP expression. (*H*) Indirect cytoskeletal cross-talk model for the mutual dependence of MRTFs and YAP. MRTF–SRF signaling influences YAP activity via mechanisms that include the potentiation of cell contractility; YAP–TEAD signaling influences MRTF activity at least in part through potentiation of TGFβ signaling. Both pathways impinge on septin–actin interaction. Likely target genes involved are shown. For discussion, see the text.

### YAP–TEAD activation of MRTF requires TGFβ signaling

Consistent with the ability of constitutively active 5SA-YAP to induce F-actin assembly, it also induced nuclear accumulation of MRTF; this was inhibited by LatB and was not induced by 5SA/S94A-YAP, indicating that it involves F-actin assembly and requires activation of YAP–TEAD target genes (Fig. 8A; Supplemental Fig. S9A). ChIP analysis showed that 5SA-YAP, but not 5SA/S94A-YAP, expression induced LatB-sensitive MRTF-A recruitment to MRTF target genes (Fig. 8B; Supplemental Fig. S9B). 5SA-YAP expression also induced phosphorylation of MRTF-A (Supplemental Fig. S9C), which reflects decreased G-actin binding (Supplemental Fig. S8A; Panayiotou et al. 2016). Consistent with these results, LatB treatment abolished 5SA-YAP activation of the MRTF reporter and MRTF-only target genes such as *Acta2* (Fig. 8C) but did not affect activation of the YAP reporter or YAP-only targets such as *Amotl2* (Fig. 8D). LatB treatment of cells expressing 5SA-YAP led to immediate inhibition of transcription at MRTF-only and shared targets but not YAP-only targets (Supplemental Fig. S9D).

We showed above that the elevated MRTF activity seen in CAFs is dependent on matrix stiffness, contractility, and TGFβ signaling (Fig. 1B–D; Supplemental Fig. S2A,B). Consistent with these data, the ability of 5SA-YAP to potentiate MRTF–SRF reporter activity and *Acta2* transcription was sensitive to the LIMK inhibitor LIMKi3, the ROCK inhibitor Y27632, and the TGFβ family receptor kinase inhibitor SB-431542, while its ability to activate the YAP–TEAD target gene *Amotl2* was not (Fig. 8E; Supplemental Fig. S9E). Strikingly, elevated TGFβ signaling in CAFs, as assessed by SMAD2 S465/S467 phosphorylation, was strongly down-regulated upon siRNA knockdown of YAP (Fig. 8F). The TGFβ component *Inhba* is expressed at high levels in CAFs (Supplemental Fig. S9F; Calvo et al. 2013) and mediates their autocrine dependence on TGFβ signaling (C Foster, unpubl.; D Miller and C Hill, pers. comm.). *Inhba* is a candidate YAP–TEAD direct target gene (Supplemental Table S2), and, in NFs, its expression was potentiated by 5SA-YAP expression (Fig. 8G).

Taken together with the results in the preceding section, these results support a model in which the potentiation of TGFβ signaling by YAP contributes to elevated MRTF–SRF activity, which in turn influences YAP–TEAD signaling through potentiation of cell contractility (Fig. 8H; see the Discussion).

## Discussion

The MRTF–SRF signaling pathway allows the coordination of gene transcription and cytoskeletal dynamics (Miralles et al. 2003; Olson and Nordheim 2010). Here we investigated the role of MRTF–SRF signaling in CAFs derived from the mouse PyMT mammary tumor model. In contrast to NFs, the MRTFs are active in CAFs and are dependent on matrix stiffness and autocrine TGFβ signaling. MRTF–SRF target gene expression is elevated and is required for CAF contractility and proinvasive properties. Strikingly, MRTF–SRF target gene expression is also dependent on the transcription coactivator YAP, which is also mechano-responsive and required for CAF proinvasive properties and vice versa even at genes that are direct targets for only one of the two pathways. MRTF and YAP can each indirectly activate the other through their ability to affect actin cytoskeletal dynamics. Our results support a model in which the mutual dependence of MRTF–SRF and YAP–TEAD arises from their ability to regulate cell contractility and TGFβ signaling (Fig. 8H). YAP functions redundantly with its relative, TAZ (Meng et al. 2016). Although activity of the latter does not appear limiting in the CAF system used here (Calvo et al. 2013), our findings suggest that MRTF–SRF activity will also influence TAZ-dependent transcription in appropriate contexts.

We found that, in contrast to NFs, a substantial proportion of mammary CAFs associated with PyMT-induced carcinoma (Calvo et al. 2013) contains nuclear MRTF and that expression of numerous MRTF–SRF target genes is concomitantly elevated. Similarly, MRTF–SRF target gene expression is elevated in tumor-proximal myCAFs (myofibroblastic CAFs) in human pancreatic ductal carcinoma (Ohlund et al. 2017). Thus, constitutively nuclear localization of MRTF and activation of MRTF–SRF target gene expression can be considered as further CAF activation markers (for discussion, see Kalluri 2016). Constitutively nuclear MRTF has been observed previously in a number of other cell types and contexts (Somogyi and Rorth 2004; Medjkane et al. 2009) and is generally taken to be indicative of increased MRTF–SRF signaling. It should be noted, however, that nuclear confinement of the MRTFs is not necessarily sufficient for activation of MRTF–SRF target gene expression unless G-actin levels are low (Vartiainen et al. 2007).

YAP-dependent gene expression is also increased in CAFs and is required for CAF contractility and proinvasive behavior (Calvo et al. 2013). Recent ChIP-seq analysis has shown that YAP–TEAD target genes encode proteins involved in proliferation, signaling, transcription, and cytoskeletal components and regulators (Stein et al. 2015; Zanconato et al. 2015). We found that the direct YAP–TEAD target gene sets contain many genes found in our previously determined MRTF–SRF gene signature (Esnault et al. 2014). These genes, which contain binding sites for both MRTF–SRF and YAP–TEAD, include several commonly used as model YAP–TEAD targets, including *Cyr61*, *Ctgf*, and *Ankrd1*. In line with our finding that YAP activity and MRTF activity are mutually dependent, we note that both MRTF–SRF and YAP–TEAD signatures defined here are significantly enriched in activated stromal gene expression signatures in both mouse tumor models and human breast, oral, and pancreatic cancers (Farmer et al. 2009; Lim et al. 2011; Moffitt et al. 2015; Ohlund et al. 2017).

We found that in CAFs, but not in normal fibroblasts, MRTF-A exhibited substrate stiffness-induced nuclear accumulation. Mechanical cues, including substrate stiffness, were shown previously to promote nuclear localization of YAP and TAZ (Wada et al. 2011; Calvo et al. 2013; Das et al. 2016; Dupont 2016). Application of force to integrins induces MRTF-A nuclear accumulation (Zhao et al. 2007), and substrate stiffness can activate it in lung fibroblasts and mesenchymal stem cells (Huang et al. 2012; Buxboim et al. 2014). It appears that development of the CAF phenotype involves increased sensitivity of YAP and MRTF to mechanical stimulation, but how this occurs remains unclear. In PDAC, contractile “myCAFs” are found close to the tumor, coexisting with a more distant “iCAF” (inflammatory CAF) population, and organoid experiments show that the myCAF phenotype requires physical contact with the tumor (Ohlund et al. 2017). This, together with the elevated expression of MRTF–SRF and YAP–TEAD target genes in myCAFs, suggests that the myCAF phenotype might be mechanically determined.

The MRTFs compete with the other cofactor family, the TCFs, for binding to SRFs (Miralles et al. 2003; Wang et al. 2004; Zaromytidou et al. 2006). In mouse embryonic fibroblasts (MEFs), simple deletion of all three TCFs substantially increases MRTF-dependent gene expression, resulting in greatly enhanced contractility and proinvasive behavior (Gualdrini et al. 2016). This phenotype reflects increased access of the MRTFs to SRFs rather than TCF-dependent transcriptional activation and supports the notion that MRTF–SRF signaling is an important contributor to the activated CAF phenotype. However, at least at the RNA level, the relative expression of the MRTFs and TCFs did not appear to differ between NFs and CAFs.

Recent experiments have demonstrated direct physical interactions between the YAP/TAZ WW domain and a conserved PPxY motif at the C terminus of myocardin family proteins, including the MRTFs (Speight et al. 2016; Yu et al. 2016; Kim et al. 2017). Our experiments with constitutively activated MRTF and YAP suggest that these interactions do not directly contribute to the transactivating functions of MRTF. Moreover, they do not appear sufficient to generate a significant ChIP signal, since, even when coincident ChIP was observed, both corresponding DNA-binding partners were also detected, consistent with either the presence of closely spaced SRF and TEAD sites or looping to remote sites. We favor the idea that rather than mediating targeting, direct MRTF–YAP interaction facilitates activation of shared targets, allowing stabilization of higher-order complexes formed between activated MRTF–SRF and YAP–TEAD bound to separate DNA regulatory elements.

While both MRTF–SRF signaling and YAP–TEAD signaling are responsive to cytoskeletal state and mechanical stress, they are differentially regulated. MRTF–SRF signaling senses G-actin levels (Miralles et al. 2003; Vartiainen et al. 2007), whereas YAP–TEAD signaling is F-actin-dependent and sensitive to cytoskeletal tension (Dupont et al. 2011; Wada et al. 2011). Moreover, even at DNA regions where both MRTF binding and YAP binding are detected, they exhibit opposite responses to the actin-binding drug CD (Miralles et al. 2003; Vartiainen et al. 2007; Wada et al. 2011; Yu et al. 2012). Nevertheless, our results show that the pathways are mutually dependent, with inhibition (or activation) of one pathway resulting in inhibition (or activation) of the other. Our results show that this interdependence requires SRF and TEAD, the DNA-binding cofactors of MRTF and YAP, and thus must involve activation of their genomic targets. Indeed, constitutively active TEAD derivatives are sufficient to induce activation of MRTF–SRF targets such as *Acta2* and *Tagln* (Ota and Sasaki 2008).

Cross-talk between MRTF–SRF and YAP–TEAD signaling involves multiple genes that influence cytoskeletal dynamics (Fig. 8H). YAP activation in CAFs is dependent on cell adhesion and contracility (Calvo et al. 2013, 2015). In normal fibroblasts, MRTF–SRF signaling potentiates YAP–TEAD signaling in an adhesion- and contractility-dependent manner. Major components of the contractile machinery in CAFs are MRTF–SRF targets, including *Acta2*, *Myl9*, and *Myh9*. MRTF–SRF signaling also potentiates expression of integrins *Itgb3* and *Itgav*, which have been implicated previously in Src-dependent YAP activation (Kaneko et al. 2014). Both contractility and YAP activation in CAFs are also dependent on the septin regulator *Cdc42ep3*, an MRTF–SRF direct target gene, which was also required for cross-talk between the MRTF–SRF and YAP–TEAD pathways. Conversely, at least one route by which YAP–TEAD signaling potentiates MRTF–SRF activity appears to be through activation of autocrine TGFβ signaling, which is likely to involve elevated expression of the YAP–TEAD target *Inhba*. Blockade of TGFβ receptor activity restores MRTF-A cytoplasmic localization in CAFs, and TGFβ signaling has been implicated previously in the myofibroblast transition (Huang et al. 2012; O'Connor et al. 2015).

We showed that the contractile and invasiveness-promoting phenotype of CAFs reflects activation of both MRTF–SRF signaling and YAP–TEAD signaling and that these two pathways are mutually dependent. The robustness of transitions between two different functional states is substantially enhanced when they are governed by interlocking positive feedback loops, such as the simultaneous activation of a pathway promoting one state and inhibition of a pathway that inactivates that state (for discussion, see Kim and Ferrell 2007; Ferrell 2008). It is tempting to speculate that the mutual dependence of the MRTF–SRF and YAP–TEAD signal pathways contributes to the stability of the CAF activated state.

## Materials and methods

### Antibodies, protein detection, and immunofluorescence

The antibodies used were phospho-Smad2 (Cell Signaling Technology, 138D4), Smad2/3 (BD, 610842), SMA (Sigma, A2547), MLC2 (Cell Signaling Technology, 3674), SRF (sc-335), phospho(T18/S19)-MLC2 (Cell Signaling Technology, 3674S), MRTF-A (sc-21558), MRTF-B (sc-47282), pan–ERK (BD, 610124), YAP (for immunofluorescence, clone 63.7, sc-101199; for ChIP, ab52771), phospho-Ser127-YAP (Cell Signaling Technology, 4911S), TEAD1/TEF-1 (BD, 610922), TEAD4/TEF3 (sc-101184), Actin (Cytoskeleton, AAN01), pan 14-3-3 (clone H-8, sc-1657), histone H3 (Abcam, ab1791); RNA Pol II CTD S5P (Covance, H14), Integrin β3 (ab119993), Integrin αV (ab179475), phospho-(Tyr416) Src (Cell Signaling Technology, 2101), Src (2109S), HA high affinity (3F10) (Roche, 11867431001), HA-peroxidase high affinity (3F10) (Roche, 12013819001), Flag-M2-peroxidase (Sigma, A-85292), and Flag (for ChIP, Sigma, F7425; for immunofluorescence, clone M2, F1804). Immunoblotting and immunofluorescence were by standard techniques. F-actin was visualized with Texas Red-X phalloidin (Invitrogen), and nuclei were visualized with DAPI.

### ChIP and RNA analysis

ChIP was as described previously (Gualdrini et al. 2016). Three independent chromatin preparations and immunoprecipitations were analyzed in each experiment with duplicate quantitative PCR (qPCR) for each using primers as in Supplemental Table S8A. Total RNA was prepared using GeneElute (Sigma) in column format. Following DNase I treatment (Ambion), cDNA synthesis used the Transcriptor first strand cDNA synthesis kit (Roche). qPCR was performed using the ABI 7900 thermocycler (Applied Biosystems) and QuantStudio QS3/QS5 with detection by SYBR Green incorporation (Invitrogen). Relative abundance of template cDNA from intronic sequences was used as a proxy for relative transcription rates and calculated using the ΔCt method, normalizing to the abundance of GAPDH cDNA. For primers, see Supplemental Table S8B.

### Gel contraction, invasion, and F-actin pelleting

Organotypic invasion assays and gel contraction assays were as described (Gualdrini et al. 2016). For inhibitor treatments and serum stimulation, cells were treated as appropriate prior to embedding and maintained in inappropriate medium during the assay. For siRNA depletions, cells were treated for 72 h prior to embedding. Twenty-four hours after plating, gel contraction was calculated as a percentage of well area using ImageJ. For F-actin pelleting, the F/G-actin separation kit (Cytoskeleton) was used according to the manufacturer's instructions.

### Cell culture, reporter assays, inhibitors, and siRNA treatments

Immortalized NFs and CAFs from the PyMT model (Calvo et al. 2013) were maintained in 10% FCS or 0.3% FCS + 1% ITS (Invitrogen) for 20 h followed by stimulation with 15% serum or 2 ng/mL TGFβ. Small-molecule treatments were 10 µM SB-143542, 20 µM CCG-203971, 1 µM LatB, 2 µM CD, 20 µM SMIFH2, 50 µM LIMKi3, 0.5 µM dasatinib, and 10 µM Y27632. Cells were treated for 12 h prior to reporter analysis and for 2 h prior to intronic RNA or ChIP analysis. Cells were transfected using Lipofectamine LTX and Plus reagent (1 µL of LTX/0.5 µL of Plus or 5 µL/2.5 µL of Plus for 24-well and six-well plates, respectively, and 30 µL of LTX/15 µL of Plus or 75 µL of LTX/37.5 µL of Plus for 10-cm and 15-cm plates). Reporter assays in 24-well plates used 20 ng of 3DA.Luc MRTF–SRF or pGL3 4xGTIIC YAP–TEAD reporter, 5 ng of Renilla ptk-RL control, and 100 ng of expression plasmid with analysis by dual-luciferase reporter assay system (Promega). Expression plasmids (15 µg per 15-cm plate) were SRF-VP16 (Miralles et al. 2003), MRTF123-1A (Vartiainen et al. 2007), pCMV-HA-5SA-YAP, and pCMV Flag-5SA/S94A-YAP (gift from Dr Ralph Gruber). For siRNA-mediated protein depletion, cells were reverse-transfected using RNAi Max reagent with analysis 72 h later; expression plasmids were transfected 48 h prior to analysis. siRNAs (20 nM) were mouse YAP1 (Dharmacon On-Target Plus, J-046247-10), human YAP1 (Dharmacon, L-012200), mouse TEAD1 (Dharmacon, L-048419-01), mouse Cdc42EP3 (Dharmacon, L-046421-01), mouse SRF (Dharmacon, J-050116-09), human SRF (Dharmacon M-009800), mouse MRTFA/B dual-targeting oligo 5′-UGGAGCUGGUGGAGAAGAA-3′ (25 nM) (Medjkane et al. 2009), human MRTF-A (L-015434), human MRTF-B (Dharmacon, M-019279), and human nontargeting pool (Dharmacon, D-001810-10-20).

### Bioinformatics

Published YAP–TEAD ChIP-seq data from IMR90 lung fibroblasts (Stein et al. 2015) were integrated with a previous Hi-C analysis of IMR90 cells (Jin et al. 2013) as described previously (Gualdrini et al. 2016). The different genome alignments used in the two studies were converted to the mm9 assembly using the University of California at Santa Cruz LiftOver tool (https://genome.ucsc.edu/cgi-bin/hgLiftOver). The final analysis revealed 725 TSSs that show physical linkage to remote YAP–TEAD sites that, together with 65 TSSs located within 2 kb of a YAP–TEAD site, give a total of 776 potential YAP–TEAD target TSSs.

Ontology analysis was performed as described previously (Gualdrini et al. 2016). Hypergeometric comparisons of target gene sets were performed in R using the phyper function. Numbers of overlapping genes were determined, and statistical significance was estimated with Bonferroni correction for multiple testing. Genes overexpressed in myCAFs (Ohlund et al. 2017) were defined as those commonly overexpressed greater than twofold in myCAFs compared with inflammatory CAFs and quiescent fibroblasts.

### Statistical analysis

Analyses were performed using GraphPad Prism software. Mean values and standard error of the mean were generated from the indicated repeats of biological experiments. *P*-values were obtained from *t*-tests with unpaired samples, with a significance threshold of *P* < 0.05.

## Supplementary Material

Supplemental Material
