# Supplementary material for: Mutual dependence of the MRTF–SRF and YAP–TEAD pathways in cancer-associated fibroblasts is indirect and mediated by cytoskeletal dynamics
Source: Genes Dev. 2017 Dec 1;31(23-24):2361–75. doi: 10.1101/gad.304501.117 (PMC5795783; doi:10.1101/gad.304501.117)
Supplement: Supplemental Material [file supp_31_23-24_2361__index.html]

Mutual dependence of the MRTF–SRF and YAP–TEAD pathways in cancer-associated fibroblasts is indirect and mediated by cytoskeletal dynamics — Supplemental Material 

# Mutual dependence of the MRTF–SRF and YAP–TEAD pathways in cancer-associated fibroblasts is indirect and mediated by cytoskeletal dynamics

## Supplemental Material

- Supplemental\_Material.pdf
- Supplemental\_Table\_S8.xlsx
- Supplemental\_Tables\_S1-S7.xlsx
