# Supplementary material for: Mutual dependence of the MRTF–SRF and YAP–TEAD pathways in cancer-associated fibroblasts is indirect and mediated by cytoskeletal dynamics
Source: Genes Dev. 2017 Dec 1;31(23-24):2361–75. doi: 10.1101/gad.304501.117 (PMC5795783; doi:10.1101/gad.304501.117)
Supplement: Supplemental Material [file supp_gad.304501.117_Supplemental_Material.pdf]

Mutual dependence of the MRTF-SRF and YAP-TEAD pathways in cancer-associated fibroblasts is indirect and mediated by cytoskeletal dynamics

Charles T Foster, Francesco Gualdrini* & Richard Treisman^

Signalling and Transcription Group, Francis Crick Institute, 1 Midland Road, London NW1 1AT, UK.

* present address: Natoli group, IFOM-IEO Campus, Via Adamello, 16-20139 Milan, Italy

^ corresponding author: Richard.Treisman@Crick.ac.uk

SUPPLEMENTAL MATERIALS

1. Supplementary Table Legends

2. Supplementary Figure Legends

3. Supplementary Figures

SUPPLEMENTARY TABLE LEGENDS

Supplemental Table S1 Genes overexpressed in CAFs versus normal fibroblasts, as determined by Illumina array analysis (Calvo et al. 2013) that are present in the MRTF-SRF stringent target gene set (Esnault et al. 2014).

Supplemental Table S2 MRTF and YAP target genes in fibroblasts. (A) MRTF-SRF direct target genes in NIH3T3 fibroblasts (Esnault 2014). (B) Candidate YAP-TEAD direct target TSS in IMR90 lung fibroblasts, integrating published IMR90 YAP-TEAD ChIP-seq (Stein et al. 2015) and Hi-C data (Jin et al. 2013). A total of 65 TSSs are within 2kb of a YAP-TEAD peak, and 725 promoters exhibit physical linkage according to Hi-C, which generates a list of 776 unique TSS. (C) Genes unique to MRTF-SRF target list ("MRTF-only" direct target genes). (D) Genes unique to candidate YAP target list ("YAP-only" direct target genes. (E) Genes in common between the candidate YAP and MRTF-SRF target genes lists ("shared" direct target genes.

Supplemental Table S3 Gene ontology analysis of all stringent MRTF-SRF target genes in NIH3T3 fibroblasts; all candidate YAP-TEAD targets listed in Table S2; and candidate MRTF-SRF specific ("MRTF-only"), YAP-TEAD-specific ("YAP-only"), and shared MRTF-SRF/YAP-TEAD targets ("shared") gene lists derived by overlapping the IMR90 and NIH3T3 data.

Supplemental Table S4 Relation between MRTF and YAP target genes in CAFs

(A) Potential YAP-TEAD targets present in the Calvo et al. (2013) Illumina dataset; (B) MRTF-SRF target genes overexpressed in CAFs that are not YAP-TEAD targets; (C) Shared MRTF-SRF/ YAP-TEAD targets target genes overexpressed in CAFs; (D) Genes overexpressed in CAFs that are potential YAP-only targets.

Supplemental Table S5. Ontology of MRTF-SRF and YAP-TEAD target genes overexpressed in CAFs.

Supplemental Table S6. Genes overexpressed in PDAC-associated myCAFs in comparison with PDAC-associated iCAFs and quiescent PSCs (Ohlund et al. 2017) that are (A) MRTF-SRF targets; (B) candidate YAP-TEAD targets; (C) MRTF-SRF-only targets; (D) shared MRTF-SRF/YAP-TEAD targets; and (E) YAP-TEAD-only targets.

Supplemental Table S7. Numbers of genes in common between the defined MRTF and YAP target signatures defined in this work and activated stromal gene expression signatures from human breast (Farmer et al. 2009), oral squamous cell (Lim et al. 2011), and pancreatic ductal cell (Moffitt et al. 2015) carcinoma were determined. Statistical significance was estimated by hypergeometric testing.

Supplemental Table S8. Primers used for qRT-PCR and ChIP analysis.

SUPPLEMENTARY FIGURE LEGENDS

Supplemental Figure S1. MRTF is activated and mechanosensitive in CAFs

(A) qPCR analysis of *Mrtf-A* and *Mrtf-B* transcripts in NF1 and CAF1 cells (B) Immunoblot analysis of αSMA/*Acta2*, MLC2/Myl9 and ERK2 (control) in NF1 and CAF1 cells with treatment with MRTF-A/B siRNA as indicated. (C) qPCR analysis of the TCF-SRF target gene, *Egr1*. (D) qPCR analysis of intronic *Acta2* and *Myl9* transcripts in CAF1 cells treated for 20h with the indicated concentrations of CCG-203971. Data are means ±SEM, n=3. (E) ChIP analysis of MRTF-A, SRF and PolIII in NF1 and CAF1 cells maintained in 0.3% FCS, with CCG203971 treatment as indicated. Data are mean ± SEM, n=3; ****, $p<0.0001$; ***, $p<0.001$. (F) Immunofluorescence microscopy of MRTF-A in normal CAF1 cells plated on stiff (50kPa) or soft (0.5kPa) polyacylamide hydrogels. Field view scale bar, 50μm.

Supplemental Figure S2. MRTF activation in CAFs requires TGFβ autocrine signals

(A) Immunoblot analysis of S465/S467-diphosphorylated Smad2, Smad2 and ERK control in NF1 and CAF1 (B) Left, immunofluorescence microscopy of MRTF-A in CAF1 cells after inhibition of TGFβ receptor signalling with SB-431542. Scale bar, 50μm. Right, quantification of 20 fields of view at 20x magnification; minimum 250 cells C, high cytoplasmic concentration; N/C equal concentration over whole cell; N, high nuclear concentration. (C) Immunoblot analysis of Smad2, S465/S467-diphosphorylated Smad2, αSMA (ACTA2), MLC2 (MYL9) and ERK2 control in NF1

and CAF1 cells with treatment with 10μM SB431542 as indicated. (D) qPCR analysis of *Acta2* and *Myl9* intronic transcripts in CAF1 cells following treatment with 10μM SB431542. Data are normalised to *Gapdh* transcripts mean ± SEM, n=3; ****, p<0.0001; ***, p<0.001. (E) Immunofluorescence microscopy of MRTF-A in NF1 cells treated with 2ng/mL TGFβ. Scale bar, 25μm. (F) qPCR analysis of *Acta2* and *Myl9* intronic transcripts in NF1 cells upon stimulation with 2ng/mL TGFβ. Quantification as in (B). ****p<0.0001 *p<0.05.

Supplemental Figure S3. Serum-stimulated activation of the MRTF-SRF reporter and target genes requires YAP and *vice versa*

NF1 cells were transfected with reporters for MRTF-SRF (A) or YAP-TEAD (D), or analysed by qPCR for intronic transcripts of MRTF-SRF (B,C), and YAP-TEAD (E,F) target genes. Cells were treated with MRTF-A/B or YAP siRNAs, and stimulated with 15% FCS for 30', as indicated. Data are mean ± SEM, n=3; ****, p<0.0001; ***, p<0.001; **, p<0.0 *, p<0.05. Note that YAP knockdown is sufficient to reduce YAP-TEAD reporter and YAP-only target gene activity to baseline, suggesting that TAZ does not play a significant role in this system.

Supplemental Figure S4. ChIP analysis demonstrates mutual dependence of MRTF and YAP DNA binding in MDA-MB231 cells

MDA-MB231 cells were treated with siRNAs against MRTF-A and MRTF-B, or YAP, or LatB (1μM, 30'), and subjected to ChIP analysis. (A) MRTF-only, YAP-only and shared target genes analysed. SRF sites, orange; TEAD sites, blue; red lines, PCR

detection probes. *Zfp37,* which binds neither MRTF-A nor YAP was used as control. (B, C) Mutual dependence of MRTF and YAP recruitment, analysed by ChIP for MRTF-A (B) and YAP (C). Data are means ± SEM, n=3 independent chromatin preparations; **, $p<0.01$; *, $p<0.05$ student's t-test.

Supplemental Figure S5. Differential sensitivity of MRTF and YAP target genes to CD

qPCR analysis of intronic transcripts MRTF-SRF and YAP-TEAD target genes in NF1 and CAF1 cells treated with 2µM CD for 30'. Data are normalised to *Gapdh* transcripts, mean ± SEM, n=3; ****, $p<0.0001$; ***, $p<0.001$; **, $p<0.01$ *, $p<0.05$..(A) MRTF-SRF-only targets (B) MRTF-SRF and YAP-TEAD shared targets; (C) YAP-TEAD-only targets.

Supplemental Figure S6. Activation of MRTF-SRF genomic targets by constitutively active MRTF does not require YAP

qPCR analysis of intronic transcripts of MRTF-SRF and YAP-TEAD target genes in NF1 cells, depleted of YAP as indicated, and transiently expressing constitutively active $MRTF^{123\text{-}1A}$. Data are normalised to *Gapdh* transcripts, mean ± SEM, n=3; *, $p<0.05$.(A) MRTF-only targets (B) YAP-only targets; (C) MRTF-SRF and YAP-TEAD shared targets. Notional contributions of YAP and MRTF activation are indicated by brackets.

Supplemental Figure S7. Activation of YAP-TEAD genomic targets by constitutively active YAP does not require MRTF

qPCR analysis of intronic transcripts of MRTF-SRF and YAP-TEAD target genes in NF1 cells, depleted of MRTF-A/B as indicated, and transiently expressing constitutively active 5SA-YAP. Data are normalised to *Gapdh* transcripts, mean ± SEM, n=3; *, p<0.05. (A) YAP-only targets (B) MRTF-only targets; (C) MRTF-SRF and YAP-TEAD shared targets. Notional contributions of YAP and MRTF activation are indicated by brackets.

Supplemental Figure S8. Constitutively active MRTF activates YAP

(A) Immunofluorescence microscopy of transiently expressed $MRTF^{123\text{-}1A}$ and 5SA-YAP in NF1 cells, stained for $MRTF^{123\text{-}1A}$ (Flag) or 5SA-YAP (HA)(top panels), F-actin (middle panels; arrowheads indicate transfected cells) or DNA (bottom panels). Scale bar, 25µm. (B) Immunofluorescence analysis MRTF-A localisation in NF1 cells with serum stimulation. (C) ChIP analysis of MDA-MB231 cells expressing $MRTF^{123\text{-}1A}$ before or after 30' LatB treatment. Data are mean ± SEM, n=3 independent chromatin preparations; ***, p<0.001; **, p<0.01; *, p<0.05. Left, ChIP of YAP at YAP-only target genes; Right, ChIP of YAP or transfected $MRTF^{123\text{-}1A}$ (using FLAG epitope tag) at shared target genes. (D) Effect of $MRTF^{123\text{-}1A}$ expression on (left) contractile and adhesion proteins, analysed by immunoblot, and (right) relative transcription of *Itgb3* and *Itgav*, assessed by qRT-PCR of intronic RNA (E) Left, relative expression of *Itgb3* and *Itgav* exonic RNA in NF1 and CAF1 cells, analysed by qRT-PCR; Right, effect of MRTF depletion on adhesion and contractile protein expression in CAFs, analysed by immunoblotting.

Supplemental Figure S9. Constitutively active YAP activates MRTF

(A) Immunofluorescence analysis of NF1 cells with or without stimulation by 15% FCS. (B) ChIP analysis of NF1 cells expressing 5SA-YAP or 5SA/S94A-YAP before or after 30' LatB treatment. Data are mean ± SEM, n=3 independent chromatin preparations; **, p<0.01; *, p<0.05. LatB does not affect binding of 5SA-YAP to target genes. Data are mean ± SEM, n=3; **, p<0.01; *, p<0.05. (C) Immunoblot analysis of the effect of 5SA-YAP on multisite phosphorylation of MRTF, as assessed by mobility reduction in 7% Tris-Acetate SDS-PAGE. (D) qPCR analysis of intronic transcripts of MRTF-SRF and YAP-TEAD target genes in NF1 cells transiently expressing 5SA-YAP, before and after a 30' LatB treatment at the indicated times after transfection. Data are normalised to *Gapdh* transcripts, mean ± SEM, n=3. Left, YAP-only target *Amotl2*; centre, MRTF-only target *Acta2*; right, shared targets *Ankrd1* and *Cyr61*. (E) Cells were transfected with 5SA-YAP, treated with the indicated inhibitors, and expression of the YAP-only target gene *Amotl2* analysed. Data are mean ± SEM, n=3. (F) Relative expression of *Inhba* in NF1 and CAF1 cells, analysed by qRT-PCR of exonic RNA.

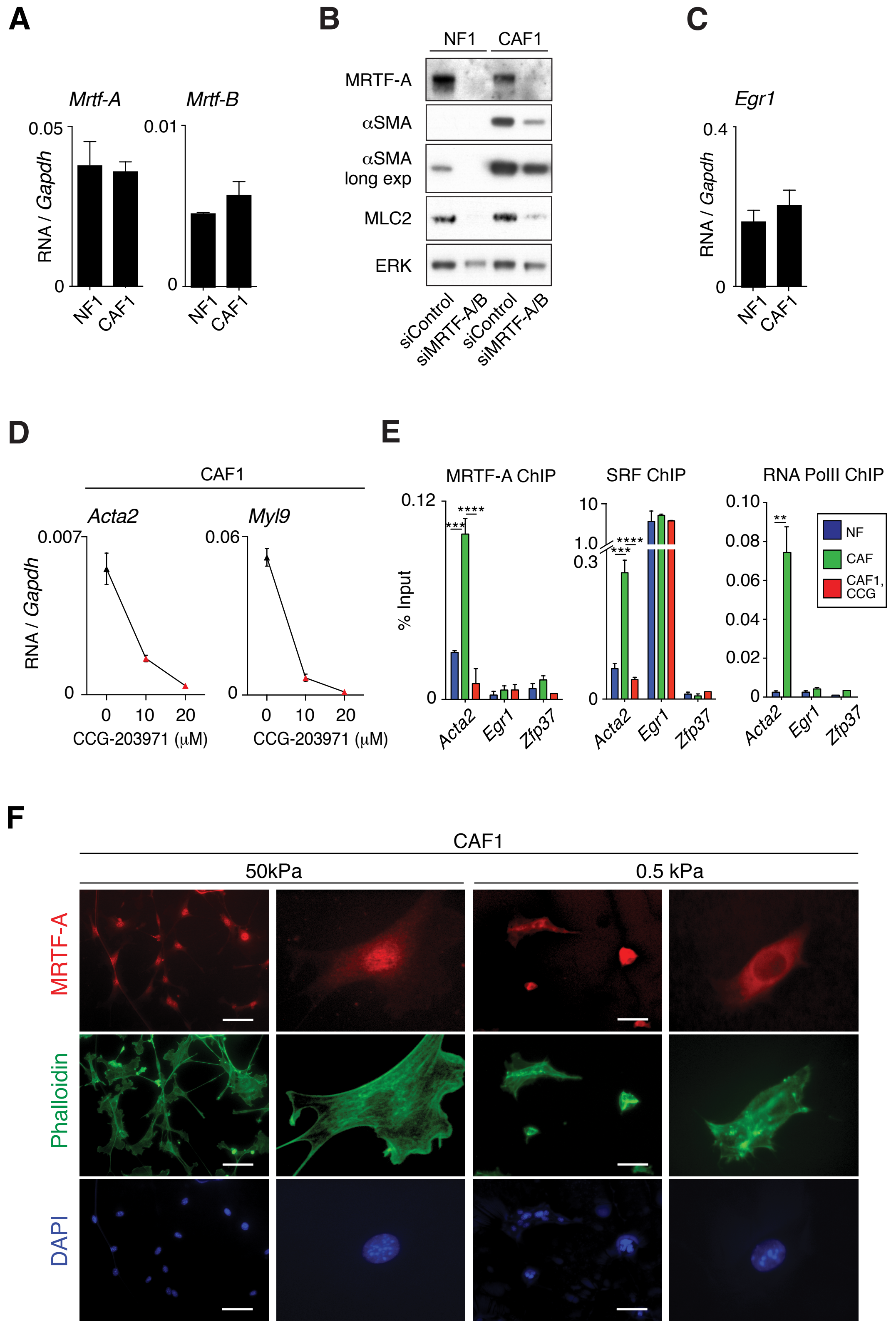
A
Mrtf-A
Mrtf-B
0.05
0.01
RNA / Gapdh
0
NF1
CAF1
B
NF1
CAF1
MRTF-A
αSMA
αSMA long exp
MLC2
ERK
siControl
siMRTF-A/B
C
Egr1
0.4
D
CAF1
Acta2
Myl9
0.007
0.06
0
10
20
CCG-203971 (μM)
E
MRTF-A ChIP
SRF ChIP
RNA PolII ChIP
0.12
10
1.0
0.3
0.10
0.08
0.06
0.04
0.02
% Input
***
****
**
NF
CAF
CAF1, CCG
Acta2
Egr1
Zfp37
F
CAF1
50kPa
0.5 kPa
MRTF-A
Phalloidin
DAPI

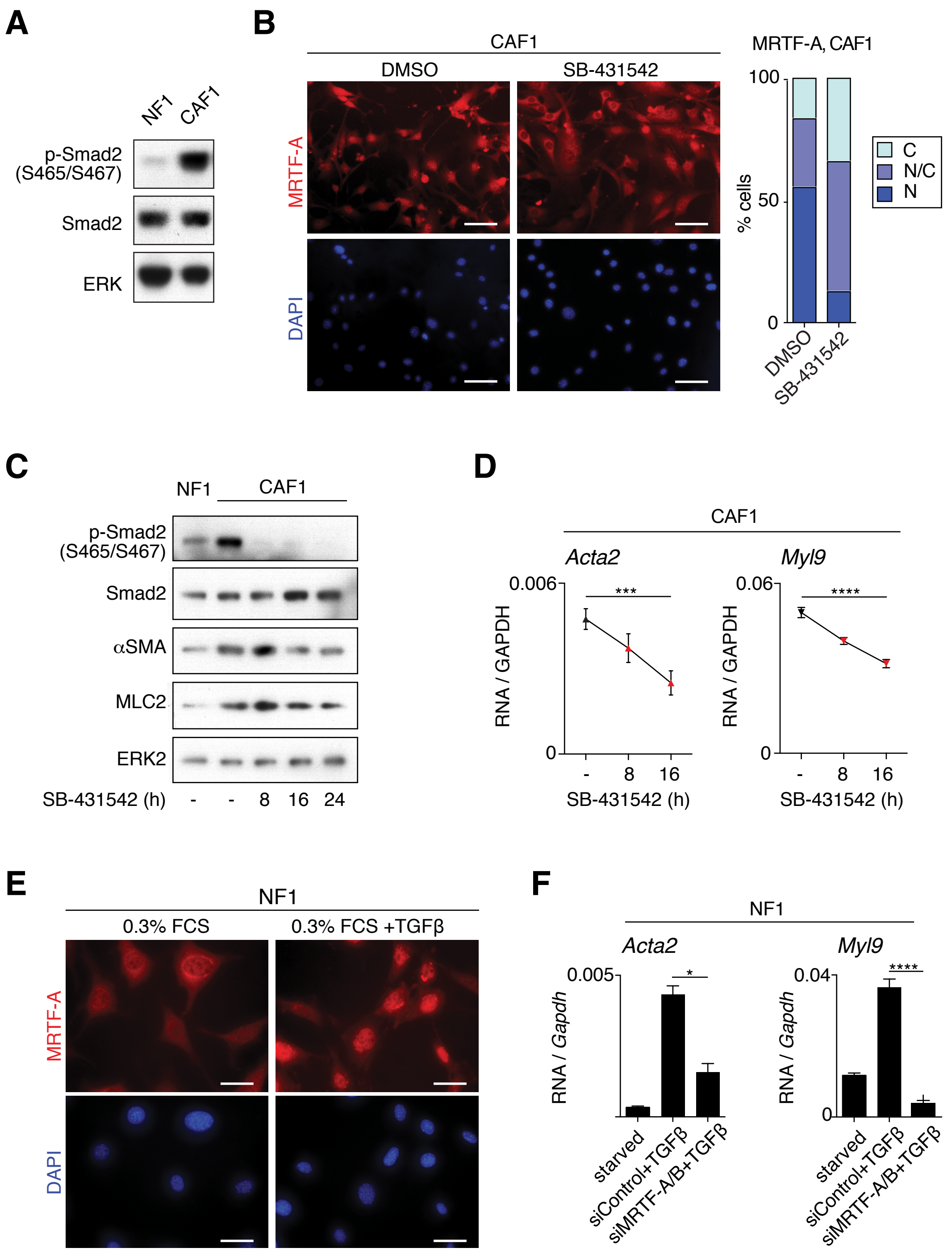
A
NF1
CAF1
p-Smad2 (S465/S467)
Smad2
ERK
B
CAF1
DMSO
SB-431542
MRTF-A
DAPI
MRTF-A, CAF1
% cells
100
50
0
C
N/C
N
C
NF1
CAF1
p-Smad2 (S465/S467)
Smad2
αSMA
MLC2
ERK2
SB-431542 (h) - - 8 16 24
D
CAF1
Acta2
Myl9
RNA / GAPDH
0.006
0.06
***
****
- 8 16
SB-431542 (h)
E
NF1
0.3% FCS
0.3% FCS +TGFβ
MRTF-A
DAPI
F
NF1
Acta2
Myl9
RNA / Gapdh
0.005
0.04
*
****
starved
siControl+TGFβ
siMRTF-A/B+TGFβ

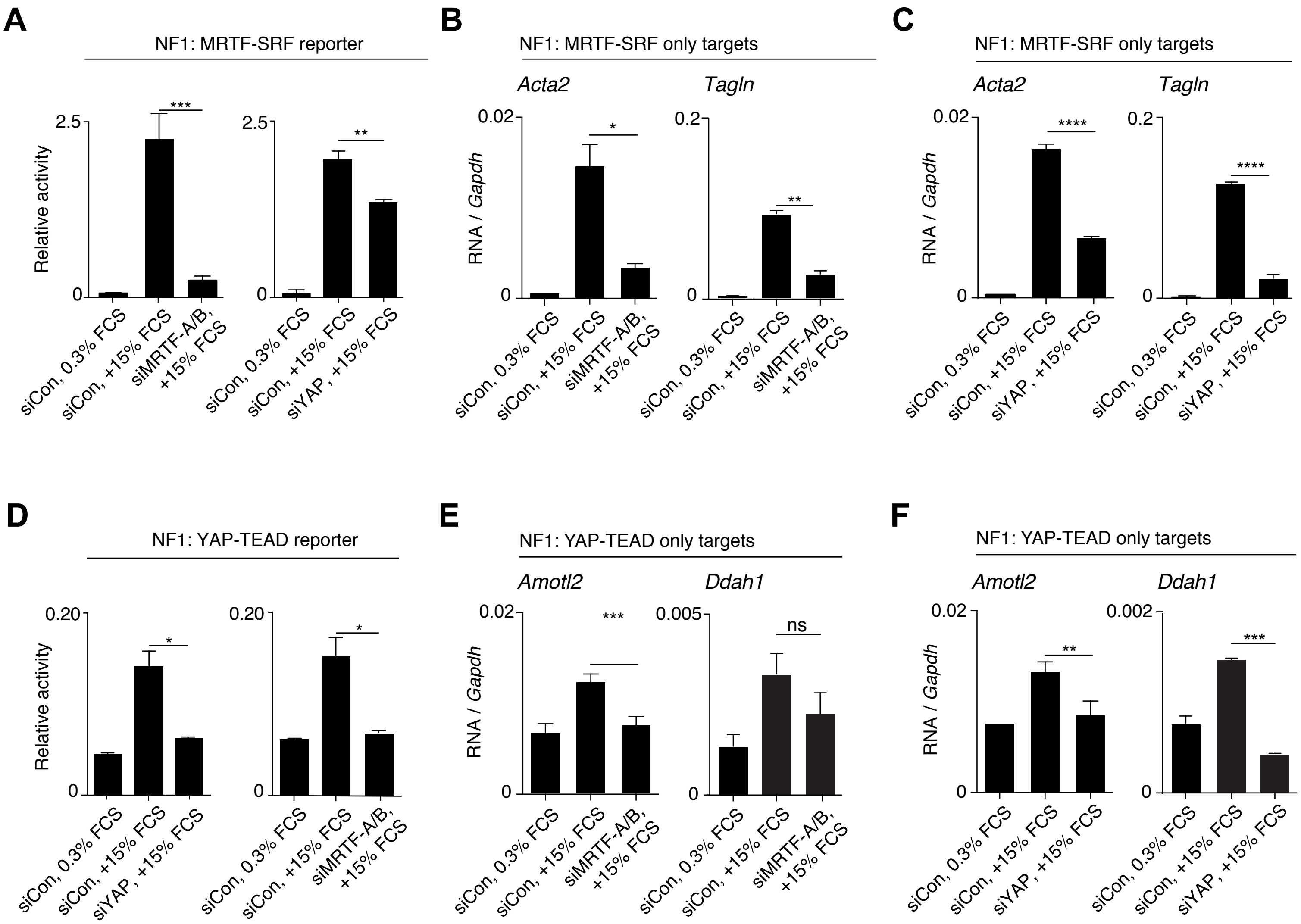
A
NF1: MRTF-SRF reporter
Relative activity
siCon, 0.3% FCS
siCon, +15% FCS
siMRTF-A/B, +15% FCS
siYAP , +15% FCS
B
NF1: MRTF-SRF only targets
Acta2
Tagln
RNA / Gapdh
C
NF1: MRTF-SRF only targets
D
NF1: YAP-TEAD reporter
E
NF1: YAP-TEAD only targets
Amotl2
Ddah1
F
NF1: YAP-TEAD only targets
ns

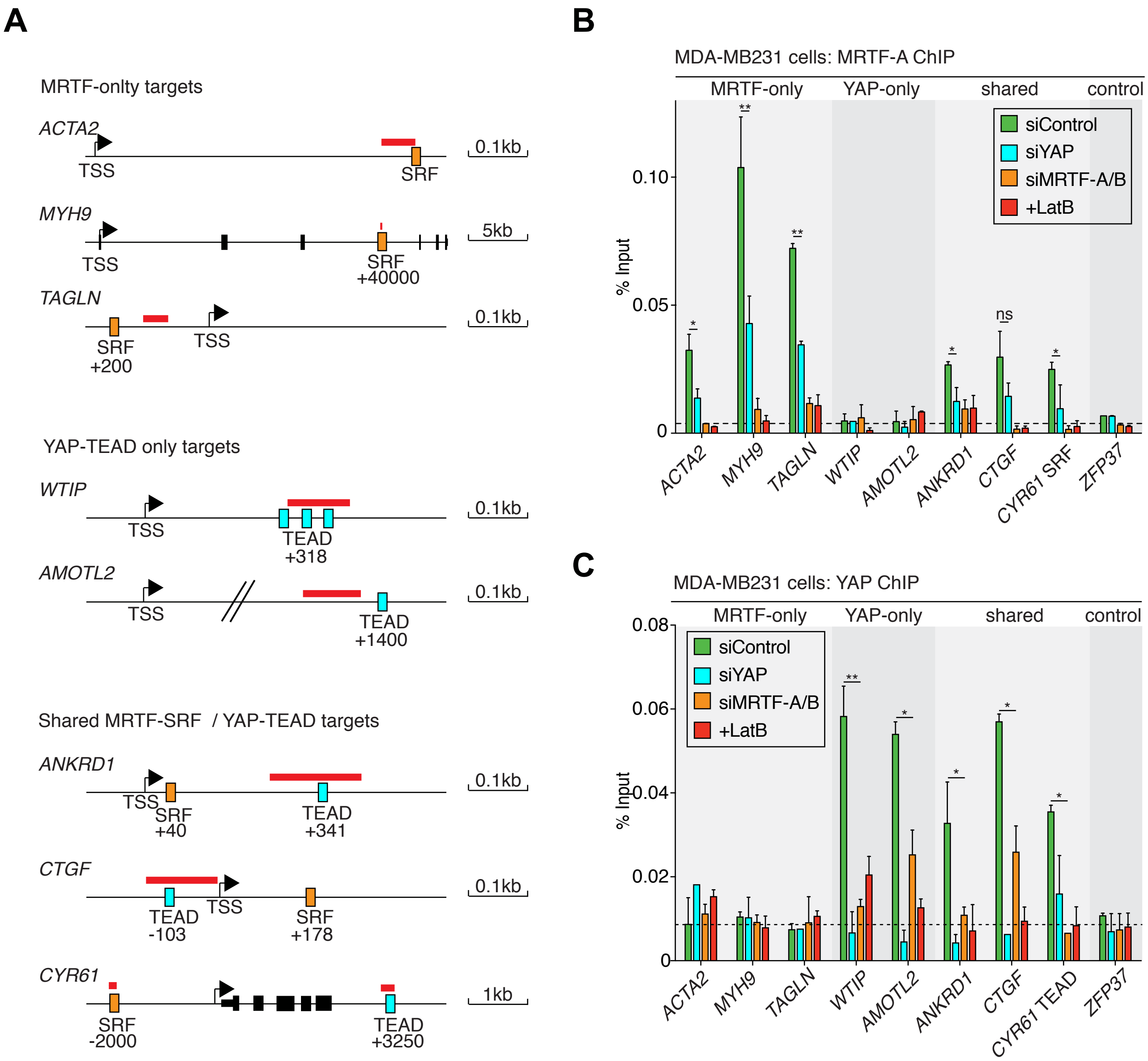
A
MRTF-onlty targets
ACTA2
TSS
SRF
0.1kb
MYH9
TSS
SRF
+40000
5kb
TAGLN
SRF
+200
TSS
0.1kb
YAP-TEAD only targets
WTIP
TSS
TEAD
+318
0.1kb
AMOTL2
TSS
TEAD
+1400
0.1kb
Shared MRTF-SRF / YAP-TEAD targets
ANKRD1
TSS
SRF
+40
TEAD
+341
0.1kb
CTGF
TEAD
-103
TSS
SRF
+178
0.1kb
CYR61
SRF
-2000
TEAD
+3250
1kb
B
MDA-MB231 cells: MRTF-A ChIP
MRTF-only
YAP-only
shared
control
siControl
siYAP
siMRTF-A/B
+LatB
% Input
0.10
0.05
0
ns
ACTA2
MYH9
TAGLN
WTIP
AMOTL2
ANKRD1
CTGF
CYR61 SRF
ZFP37
C
MDA-MB231 cells: YAP ChIP
MRTF-only
YAP-only
shared
control
siControl
siYAP
siMRTF-A/B
+LatB
% Input
0.08
0.06
0.04
0.02
0
ACTA2
MYH9
TAGLN
WTIP
AMOTL2
ANKRD1
CTGF
CYR61 TEAD
ZFP37

A **MRTF-only targets (intronic RNA)**

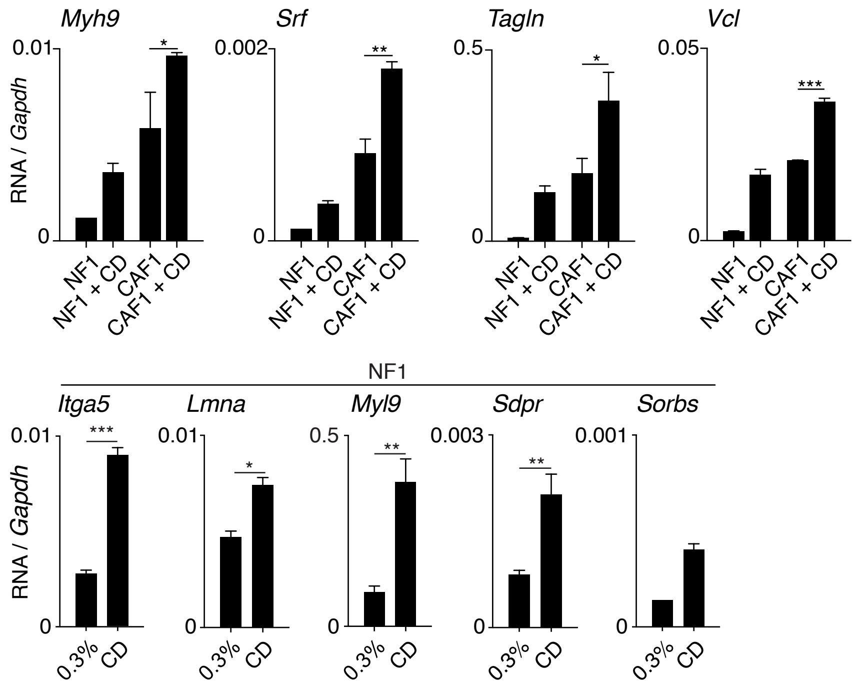

B **Shared targets (intronic RNA)**

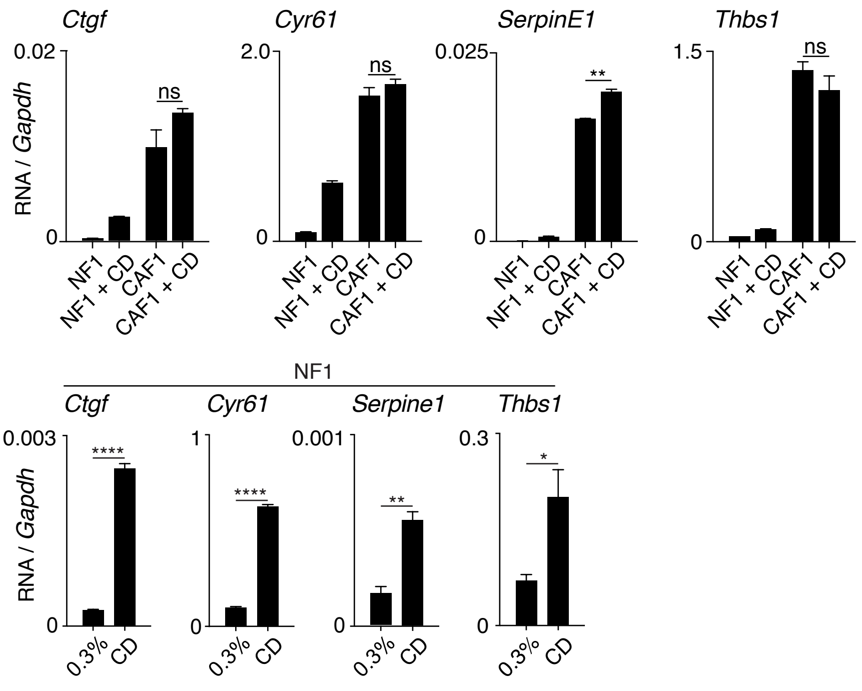

D **YAP-only targets (intronic RNA)**

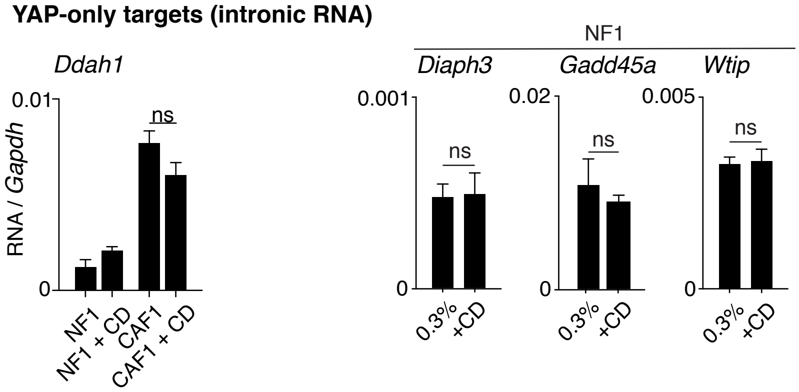

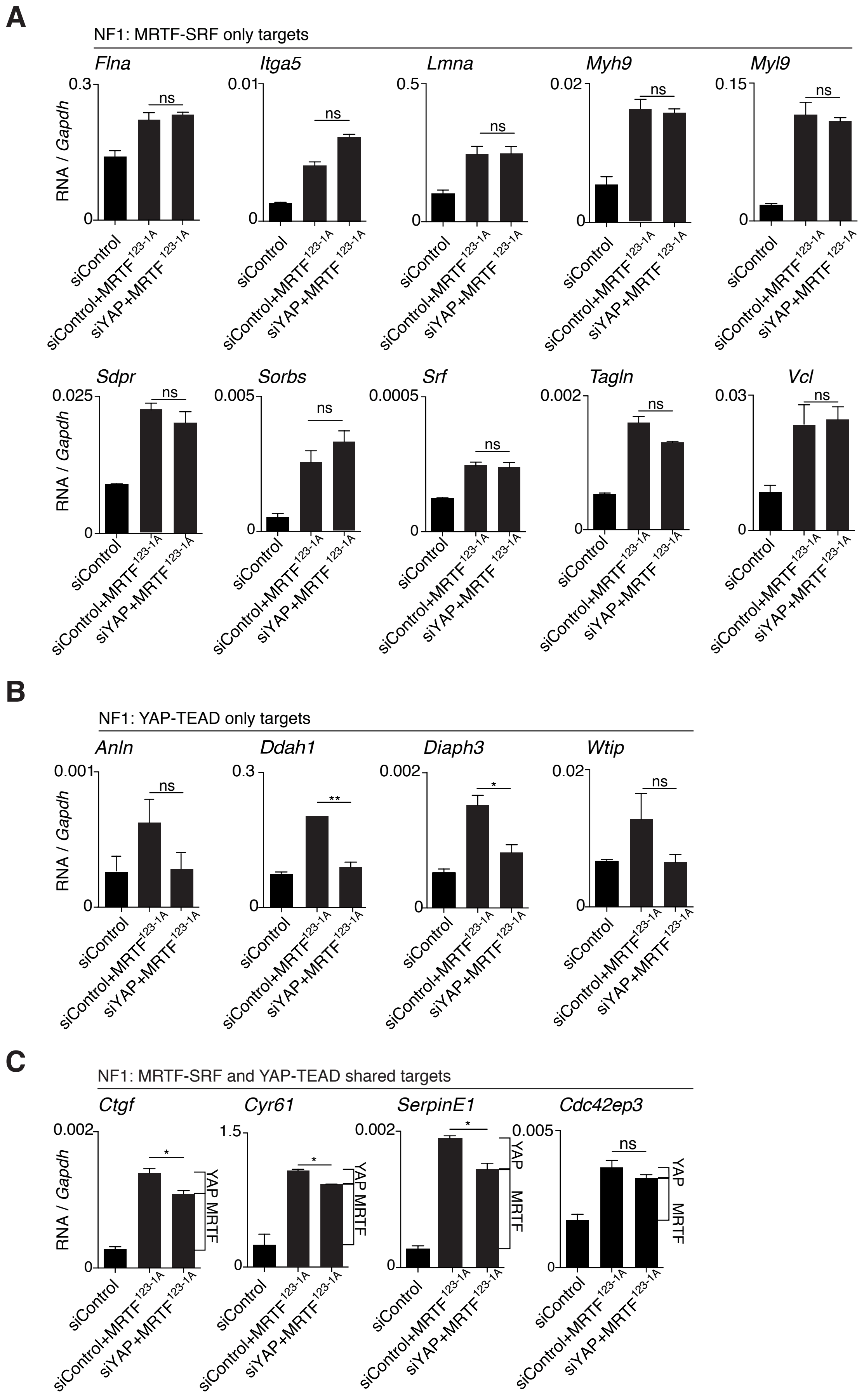
A
NF1: MRTF-SRF only targets
Flna
Itga5
Lmna
Myh9
Myl9
Sdpr
Sorbs
Srf
Tagln
Vcl
RNA / Gapdh
siControl
siControl+MRTF 123-1A
siYAP+MRTF 123-1A
ns
B
NF1: YAP-TEAD only targets
Anln
Ddah1
Diaph3
Wtip
**
*
C
NF1: MRTF-SRF and YAP-TEAD shared targets
Ctgf
Cyr61
SerpinE1
Cdc42ep3
YAP
MRTF

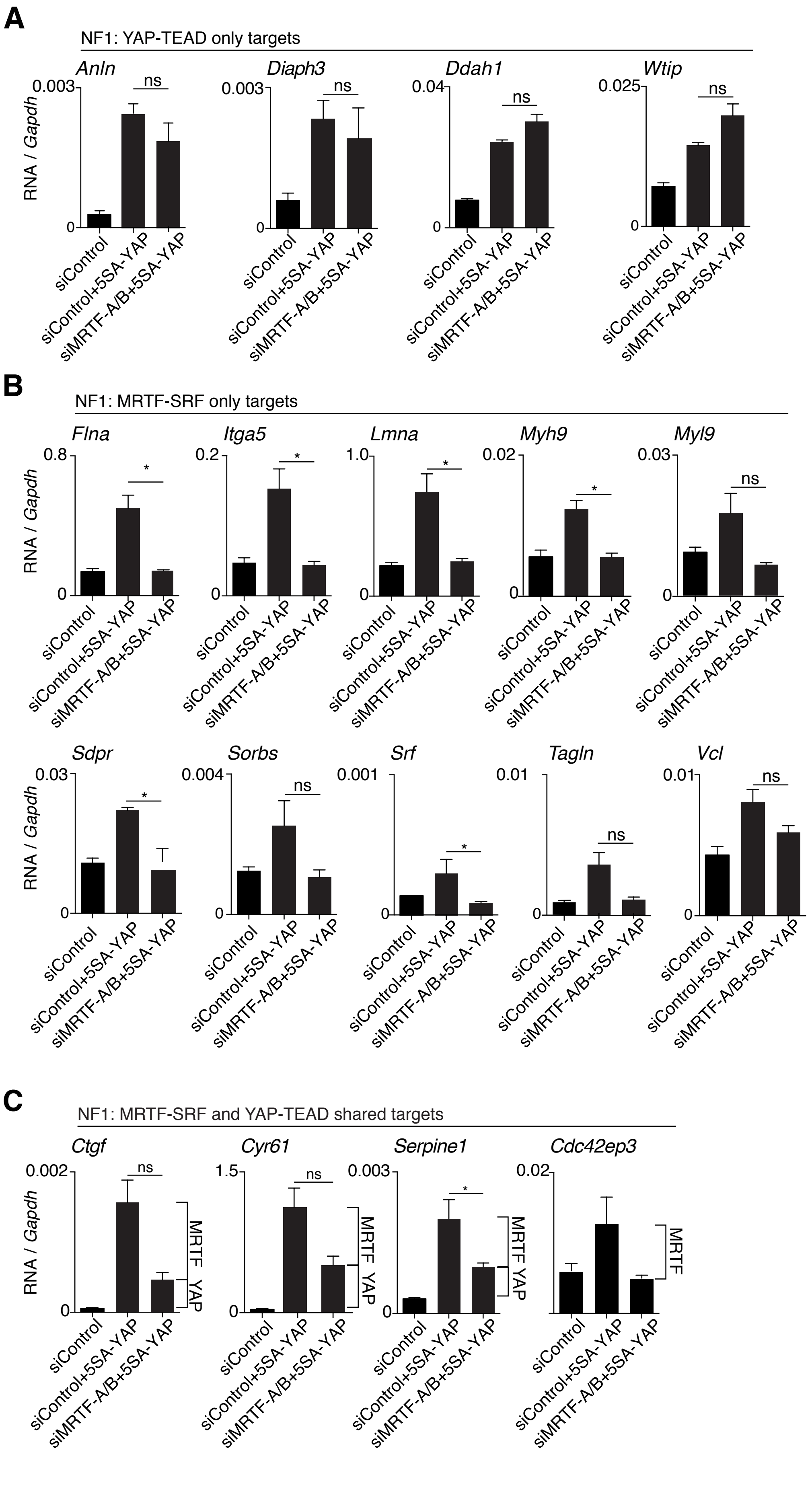
A
NF1: YAP-TEAD only targets
Anln
Diaph3
Ddah1
Wtip
RNA / Gapdh
0.003
0.04
0.025
0
ns
siControl
siControl+5SA-YAP
siMRTF-A/B+5SA-YAP
B
NF1: MRTF-SRF only targets
Flna
Itga5
Lmna
Myh9
Myl9
0.8
0.2
1.0
0.02
0.03
*
Sdpr
Sorbs
Srf
Tagln
Vcl
0.004
0.001
0.01
C
NF1: MRTF-SRF and YAP-TEAD shared targets
Ctgf
Cyr61
Serpine1
Cdc42ep3
0.002
1.5
MRTF
YAP

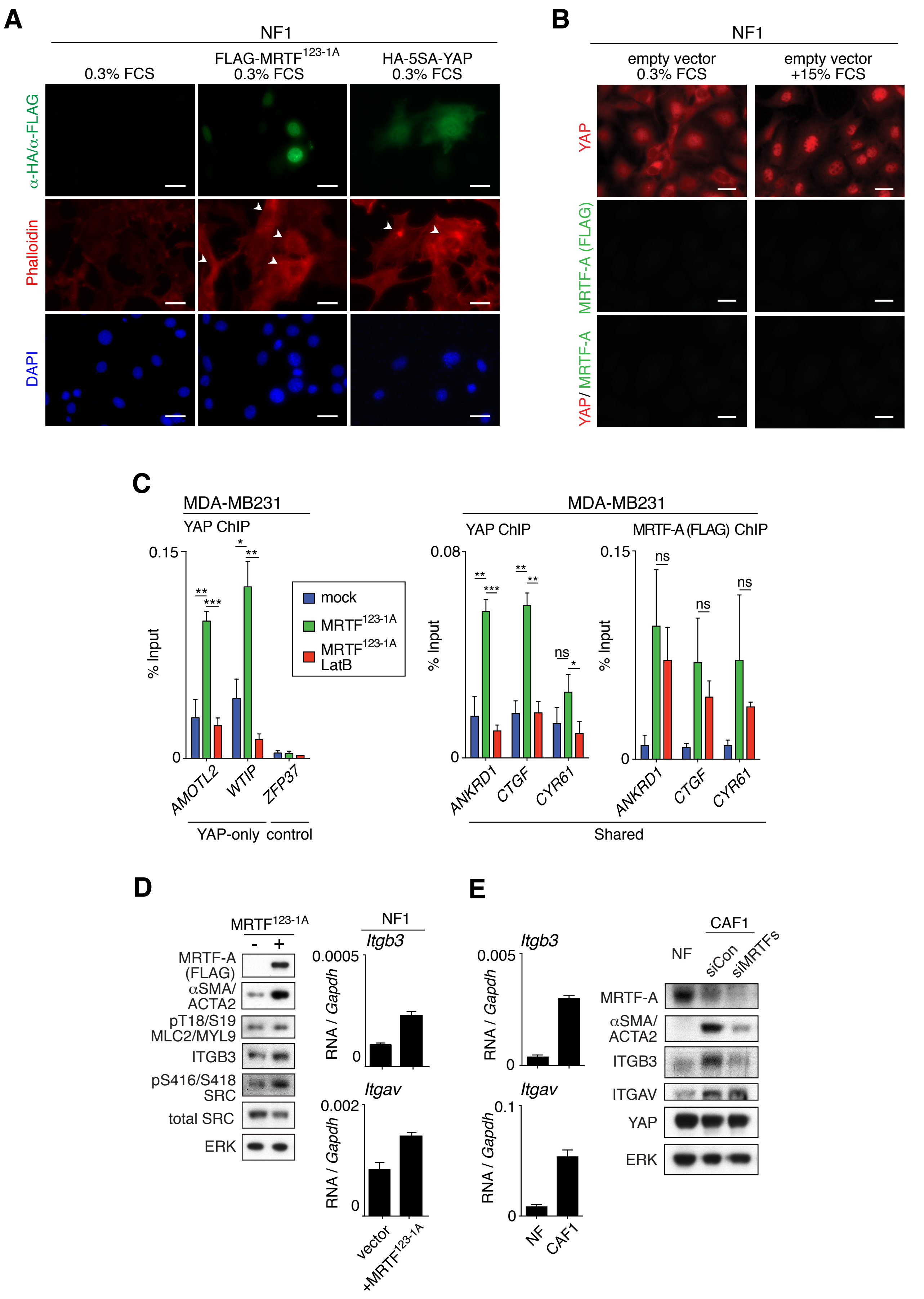
A
NF1
0.3% FCS
FLAG-MRTF123-1A 0.3% FCS
HA-5SA-YAP 0.3% FCS
α-HA/α-FLAG
Phalloidin
DAPI
B
NF1
empty vector 0.3% FCS
empty vector +15% FCS
YAP
MRTF-A (FLAG)
YAP/ MRTF-A
C
MDA-MB231
YAP ChIP
MRTF-A (FLAG) ChIP
% Input
mock
MRTF123-1A
MRTF123-1A LatB
AMOTL2
WTIP
ZFP37
ANKRD1
CTGF
CYR61
YAP-only
control
Shared
ns
D
MRTF123-1A
MRTF-A (FLAG)
αSMA/ ACTA2
pT18/S19 MLC2/MYL9
ITGB3
pS416/S418 SRC
total SRC
ERK
NF1
Itgb3
Itgav
RNA / Gapdh
vector
+MRTF123-1A
E
NF
CAF1
siCon
siMRTFs
MRTF-A
ITGAV
YAP

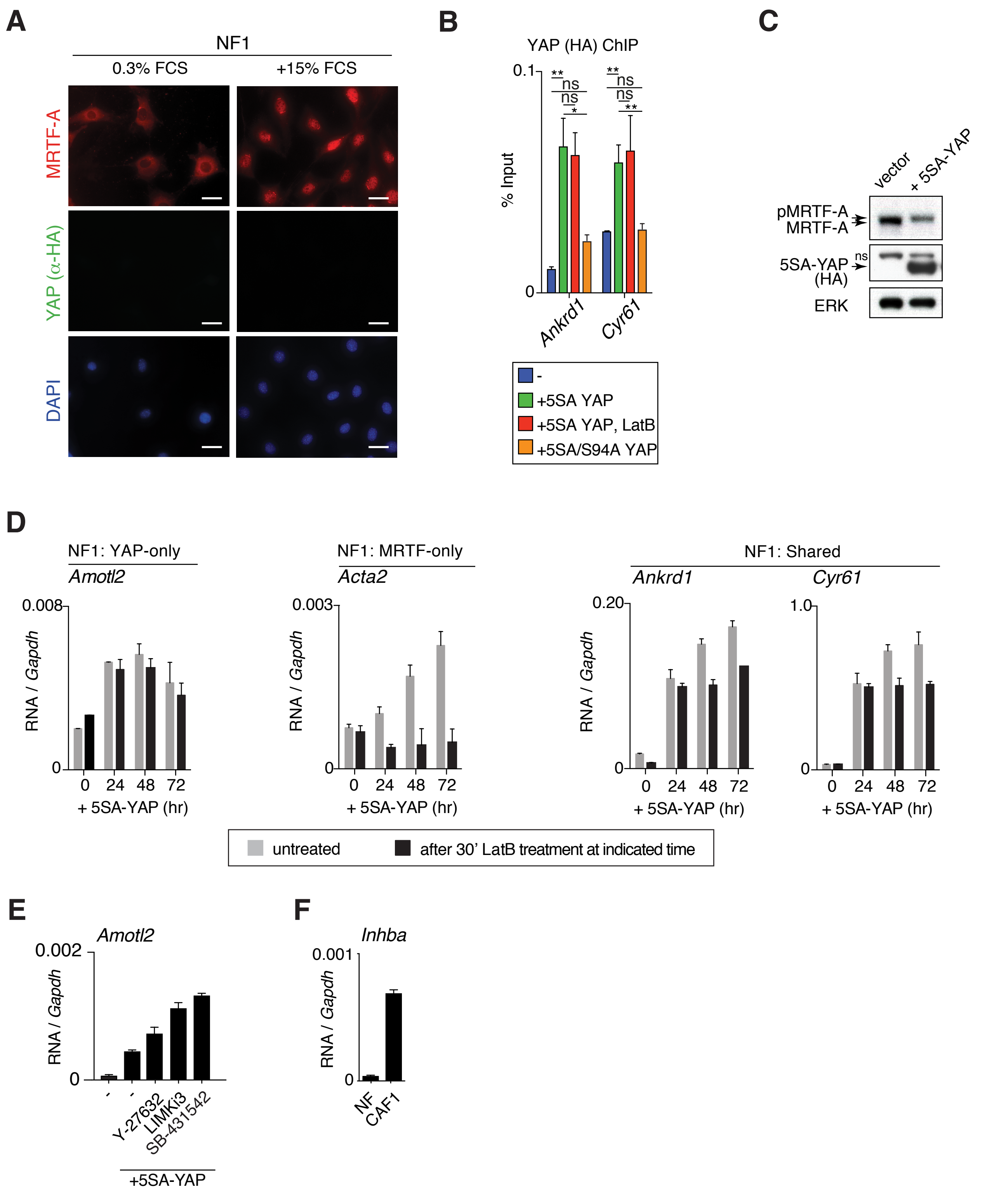
A
NF1
0.3% FCS
+15% FCS
MRTF-A
YAP (α-HA)
DAPI
B
YAP (HA) ChIP
% Input
0.1
0
Ankrd1
Cyr61
-
+5SA YAP
+5SA YAP, LatB
+5SA/S94A YAP
C
vector
+ 5SA-YAP
pMRTF-A
MRTF-A
5SA-YAP (HA)
ns
ERK
D
NF1: YAP-only
Amotl2
NF1: MRTF-only
Acta2
NF1: Shared
Ankrd1
Cyr61
RNA / Gapdh
0.008
0.003
0.20
1.0
0 24 48 72
+ 5SA-YAP (hr)
untreated
after 30' LatB treatment at indicated time
E
Amotl2
0.002
Y-27632
LIMKi3
SB-431542
+5SA-YAP
F
Inhba
0.001
NF
CAF1
